# Supplementary material for: Cotranslational Protein Folding inside the Ribosome Exit Tunnel
Source: Cell Rep. 2015 Aug 28;12(10):1533–40. doi: 10.1016/j.celrep.2015.07.065 (PMC4571824; doi:10.1016/j.celrep.2015.07.065)
Supplement: Document S1. Supplemental Experimental Procedures and Figures S1–S4 [file mmc1.pdf]

Cell Reports

Supplemental Information

Cotranslational Protein Folding inside the Ribosome Exit Tunnel

Ola B. Nilsson, Rickard Hedman, Jacopo Marino, Stephan Wickles, Lukas Bischoff, Magnus Johansson, Annika Müller-Lucks, Fabio Trovato, Joseph D. Puglisi, Edward P. O’Brien, Roland Beckmann, and Gunnar von Heijne

a

```
MANRSFIYEPFQIPSGSMMPTLNSTDFILVEKFAYGIKDPIYQKTLIETGHPKRGDIVVFKYPEDPKLDYIKRAVGLPGDKVTY
DPVSKELTIQPGCSSGQACENALPVTYSNVEPSDFVQTFSRRNGGEATSGFFEVPKQETKENGIRLSETSGSGSKPYPCGLCNR
CFTRRDLLIRHAQKIHSGNSGSGVPGQQNATWIVPPGQYFMMGDWMSSFSTPVWISQAQGIRAGPGSSDKQEGEWPTGLRLSRI
GGIH
```

b

Ribosome (~30 aa)

```
                                                                      L
                              GSGSKPYPCGLCNRCFTRRDLLIRHAQKIHSGNFSTPVWISQAQGIRAGP 17
                           GSGSKPYPCGLCNRCFTRRDLLIRHAQKIHSGNMSSFSTPVWISQAQGIRAGP 20
                         GSGSKPYPCGLCNRCFTRRDLLIRHAQKIHSGNSGMSSFSTPVWISQAQGIRAGP 22
                        GSGSKPYPCGLCNRCFTRRDLLIRHAQKIHSGNSGSMSSFSTPVWISQAQGIRAGP 23
                       GSGSKPYPCGLCNRCFTRRDLLIRHAQKIHSGNSGSGMSSFSTPVWISQAQGIRAGP 24
                      GSGSKPYPCGLCNRCFTRRDLLIRHAQKIHSGNSGSGVMSSFSTPVWISQAQGIRAGP 25
                     GSGSKPYPCGLCNRCFTRRDLLIRHAQKIHSGNSGSGVPMSSFSTPVWISQAQGIRAGP 26
                    GSGSKPYPCGLCNRCFTRRDLLIRHAQKIHSGNSGSGVPGMSSFSTPVWISQAQGIRAGP 27
                   GSGSKPYPCGLCNRCFTRRDLLIRHAQKIHSGNSGSGVPGWMSSFSTPVWISQAQGIRAGP 28
                  GSGSKPYPCGLCNRCFTRRDLLIRHAQKIHSGNSGSGVPGQWMSSFSTPVWISQAQGIRAGP 29
                 GSGSKPYPCGLCNRCFTRRDLLIRHAQKIHSGNSGSGVPGQQWMSSFSTPVWISQAQGIRAGP 30
                GSGSKPYPCGLCNRCFTRRDLLIRHAQKIHSGNSGSGVPGQQNWMSSFSTPVWISQAQGIRAGP 31
               GSGSKPYPCGLCNRCFTRRDLLIRHAQKIHSGNSGSGVPGQQNAWMSSFSTPVWISQAQGIRAGP 32
              GSGSKPYPCGLCNRCFTRRDLLIRHAQKIHSGNSGSGVPGQQNATWMSSFSTPVWISQAQGIRAGP 33
           GSGSKPYPCGLCNRCFTRRDLLIRHAQKIHSGNSGSGVPGQQNATWAIWMSSFSTPVWISQAQGIRAGP 36
          GSGSKPYPCGLCNRCFTRRDLLIRHAQKIHSGNSGSGVPGQQNATWIVPWMSSFSTPVWISQAQGIRAGP 37
        GSGSKPYPCGLCNRCFTRRDLLIRHAQKIHSGNSGSGVPGQQNATWIVPPGWMSSFSTPVWISQAQGIRAGP 39
      GSGSKPYPCGLCNRCFTRRDLLIRHAQKIHSGNSGSGVPGQQNATWIVPPGQYWMSSFSTPVWISQAQGIRAGP 41
    GSGSKPYPCGLCNRCFTRRDLLIRHAQKIHSGNSGSGVPGQQNATWIVPPGQYFMWMSSFSTPVWISQAQGIRAGP 43
 GSGSKPYPCGLCNRCFTRRDLLIRHAQKIHSGNSGSGVPGQQNATWIVPPGQYFMMGDWMSSFSTPVWISQAQGIRAGP 46
```

Figure S1, related to Fig. 1. (a) Seqeunce of the full ADR1a-SecM(*L*=46) construct. ADR1a is in red, the SecM AP in green, residues from the periplasmic domain of *E. coli* LepB in black, and linker residues generated during construction in grey. (b) Sequences of the ADR1a-AP part of all constructs analyzed in Fig. 1d. Zn^{2+}-binding residues are highligthed in blue.

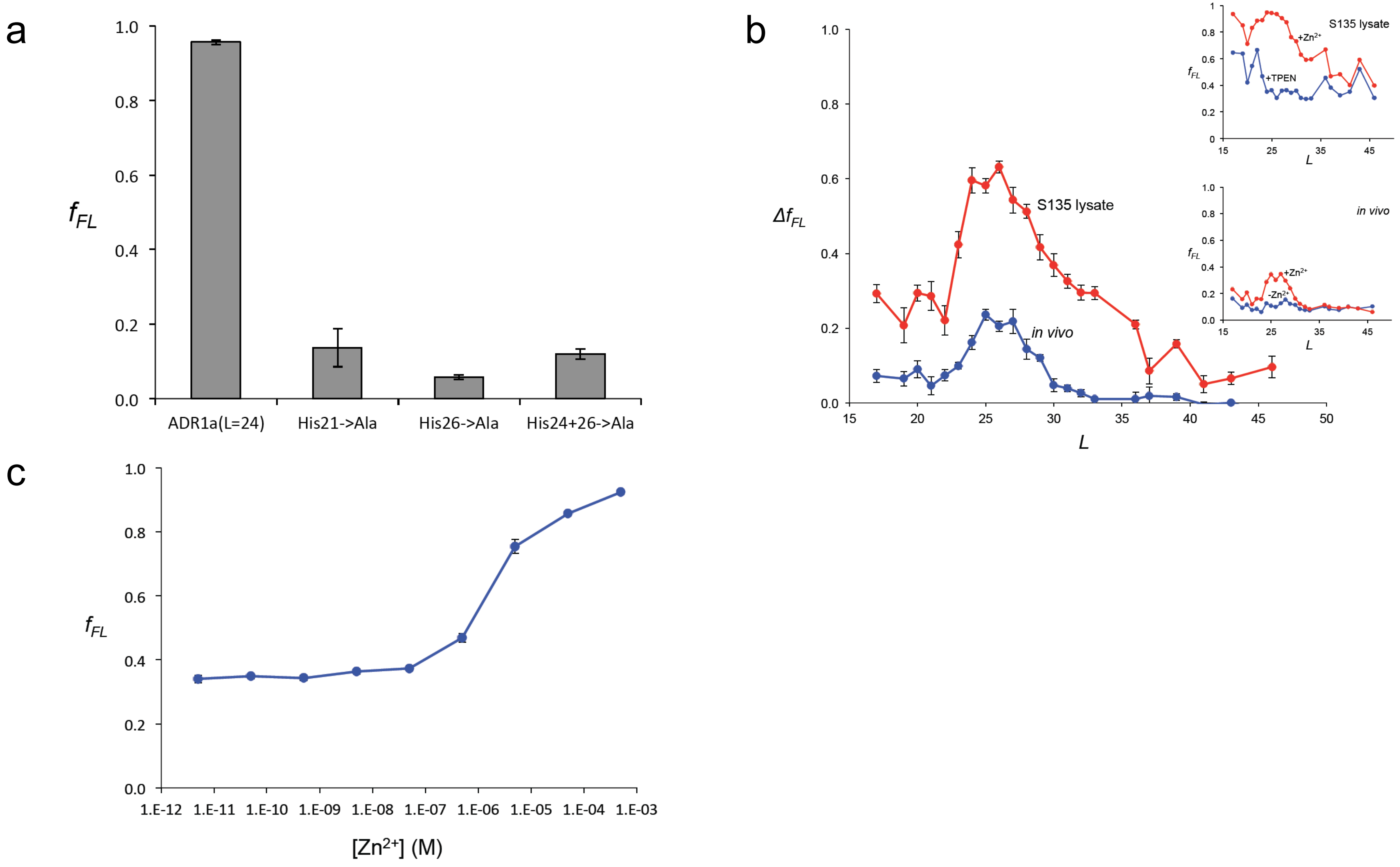

Figure S2, related to Fig.1. (a) Mutation of the two Zn^{2+}-binding His residues in the ADR1a-SecM(*L*=24) construct to Ala reduces f_{FL} to background values. Standard errors (s.e.m.) are indicated. (b) Zn^{2+}-induced folding of ADR1a in an *E.coli* S135 lysate (red) and *in vivo* (blue). The difference in f_{FL} between reactions run in the presence (500 µM) and absence of Zn^{2+} is shown. Standard errors (s.e.m.) are indicated. Insets show the individual f_{FL} profiles $\pm Zn^{2+}$ for the S135 lysate (top) and *in vivo* (bottom). (c) Zn^{2+} titration of folding of ADR1a(*L*=26) translated in *E. coli* S135 lysate. Standard errors (s.e.m.) are indicated.

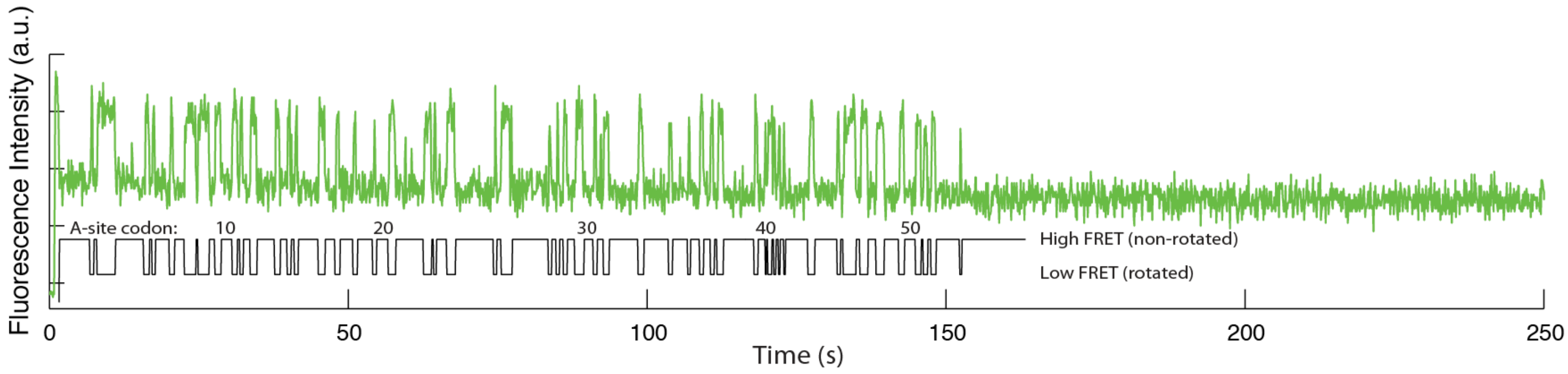

Figure S3, related to Fig.2. Time-trace showing one ribosome translating the ADR1a-SecM ORF in the absence of Zn^{2+}. The ribosome stalls at codon 54.

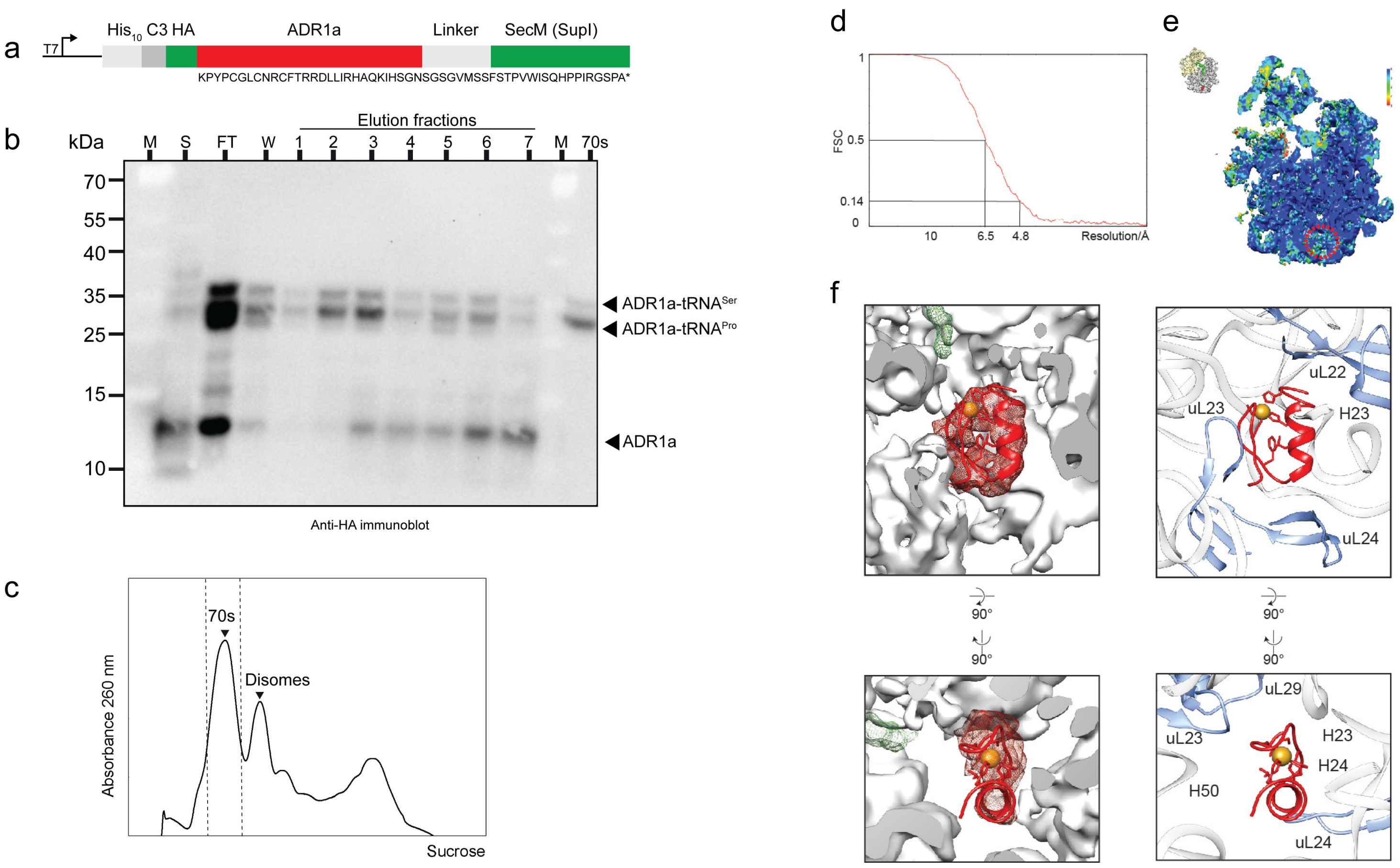

Figure S4, related to Fig.4. Purification of the ADR1a-SecM(*Ms*-Sup1; *L*=25) ribosome-nascent chain complex, and resolution of ADR1-RNCs and local environment of the ADR1 domain in the ribsomal tunnel. (a) Construct for programming and affinity purification of ADR1a-SecM(*Ms*-Sup1; *L*=25) RNCs for cryo-EM analysis. (b) Analysis of RNC purification by Western-blot using anti-HA antibody and 12% Nu-Page gel separation of the supernatant fraction after ultracentrifugation (S), the flow through fraction after Ni-NTA column (FT), the wash fraction (W), and elution fractions. Bands corresponding to Ser- and Pro-peptidyl-tRNA-ADR1a complexes and the free peptide corresponding to ADR1a are indicated. (c) UV profile after sucrose density gradient centrifugation of affinity purified RNCs. Dashed lines indicate the fraction collected for by cryo-EM analysis. (d) FSC of the RNC-ADR1a-SecM(*Ms*-Sup1;*L*=25) reconstruction indicating an average resolution of 4.8 Å. (e) Local resolution (Kucukelbir et al., 2014) shown on a cut density, revealing resolution for the ADR1a domain similar to the ribosome. (f) Close-up of the tunnel with the structure of the ADR1a domain fitted as a rigid body depicted in red. The identities of nearby ribosomal proteins and rRNA are indicated.

SUPPLEMENTAL EXPERIMENTAL PROCEDURES

Enzymes and Chemicals

Unless otherwise stated, all enzymes were obtained from Thermo Scientific (Waltham, MA, USA) and New England Biolabs (Ipswich, MA, USA). Oligonucleotides were from Eurofins MWG Operon (Ebersberg, Germany). PUREfrex™ cell-free translation system was purchased from BioNordika (Stockholm, Sweden) and DNA/RNA purification kits were obtained from Qiagen (Hilden, Germany). [^{35}S]-methionine was from PerkinElmer (Waltham, MA, USA). All other reagents were from Sigma-Aldrich (St. Louis, MO, USA).

DNA Manipulations

All ADR1a constructs were generated from the previously described pING1 plasmid carrying a truncated *lepB* gene containing a [6L,13A] H segment insert and the *Escherichia coli* SecM arrest peptide, FSTPVWISQAQGIRAGP, under the control of an arabinose-inducible promoter (Ismail et al., 2012). A soluble, non-membrane targeted LepB derivative was generated by a deletion of codon 4-77 using PCR, corresponding to the removal of transmembrane segment 1 and 2. The resulting plasmid was digested with *SpeI* and *KpnI* to release the [6L,13A] segment, and oligonucleotides corresponding to a GSGS-flanked ADR1a domain (GSGS-KPYPCGLCNRCFTRRDLLIRHAQKIHSGN-SGSG) were ligated in its place. Shorter linker lengths, *L*, between ADR1a and the arrest peptide were generated by shortening the linker from its N-terminal end by PCR as previously described (Ismail et al., 2012). Site-directed mutagenesis was performed to generate constructs with the non-functional FSTPVWISQAQGIRAG<u>A</u> arrest peptide and constructs with ADR1a domains where either one or both of the underlined, Zn^{2+}-binding His residues in the

sequence KPYPCGLCNRCFTRRDLLIRHAQKIHSGN were changed to Ala. For RNA transcription using the T7 promoter, all constructs were subcloned into pET19b (Novagen, Madison, WI, USA) using *NcoI* and *BamHI*.

***In Vitro* Transcription and Translation for Measurements of f_{FL}.**

In vitro transcription was performed with T7 RNA polymerase according to the manufacturer’s protocol (Promega) using PCR products as templates for the generation of truncated nascent chains. RNA obtained was purified using RNeasy Mini Kit (Qiagen). Translation was performed in the commercially available PUREfrex™ system (Shimizu et al., 2005) and in a S135 *E. coli* extract. The S135 cell extract was prepared as previously described (Schwarz et al., 2007). To obtain an essentially Zn^{2+}-free S135 cell extract for the Zn^{2+}-titration assay (Fig. S2c), we added three additional dialysis steps to the normal protocol, where 50 µM of the Zn^{2+} chelator N,N,N,N-Tetrakis(2-pyridylmethyl)ethylenediamine (TPEN) was added to the normal dialysis buffer. *In vitro* translation in the PURE system was performed according to the manufacturer’s protocol. *In vitro* translation in the S135 cell extracts was performed essentially as described (Welte et al., 2012). Translation was carried out in a buffer containing 40 mM Tris pH 7.5, 140 mM KOAc, 10 mM Mg^{2+} and 0.8 mM Spermidine. The translation reactions were supplemented with either 50 µM (PURE) or 500 µM (S135 extract) zinc acetate, or 50 µM TPEN. Synthesis of [^{35}S]-Met labeled polypeptides in both translation systems was performed at 37 °C with shaking at 500 rpm for 15 minutes. The reaction was stopped by the addition of equal volume of 10% ice-cold TCA and the samples were incubated on ice for 30 min and spun for 5 min at 20,800 g at 4°C. Pellets were dissolved in sample buffer and treated with RNase A (400 µg ml^{-1}) for 15 min at 37 °C before the samples were resolved by SDS-PAGE. Gels were visualized using a Fuji FLA-3000 phosphoimager and the

ImageGauge V4.23 software. Quantification of protein bands was performed using the QtiPlot 0.9.7.10 software. f_{FL} values were calculated as $f_{FL} = I_{FL}/(I_{FL}+I_A)$, where I_{FL} is the intensity of the band corresponding to the full-length protein and I_A is the intensity of the band corresponding to the arrested form of the protein, *c.f.*, Fig. 1c. Experiments were repeated three times, and standard errors (s.e.m.) were calculated.

In Vivo Pulse-Labeling Analysis

E. coli MC1061 cells bearing the respective plasmids were grown overnight at 37°C in M9 minimal media supplemented with 19 natural amino acids (1 μg ml^{-1}; no Met), 100 μg ml^{-1} thiamine, 0.1 mM $CaCl_2$, 2 mM $MgSO_4$, 0.4% (w/v) fructose, and 100 μg ml^{-1} ampicillin. Cultures were back-diluted to OD_{600} = 0.1, grown to OD_{600} = 0.35 and split into two subcultures. Expression of the ADR1a-SecM constructs was induced with 0.2% (w/v) arabinose for 5 min. $ZnCl_2$ was added to one of the two subcultures to a final concentration of 0.5 mM at the point of induction using a mixture of arabinose and $ZnCl_2$. Cells were then pulsed-labeled with [^{35}S]-Met for 2 min at 37°C before being added to an equal volume of 20% ice-cold trichloroacetic acid (TCA). Samples were incubated on ice for 30 min and spun for 5 min at 20,800 g at 4°C. Pellets were washed with cold acetone, spun again for 5 min at 4°C, and subsequently solubilized in Tris-SDS solution (10 mM Tris-Cl, pH 7.5, 2% SDS) at 95°C for 10 min. Samples were spun for 5 min at room temperature and the lysate was used for immunoprecipitation using LepB antisera. The samples were resolved by SDS-PAGE and quantitated as described above. Experiments were repeated three times using independent culture incubations, and standard errors (s.e.m.) were calculated.

Single-Ribosome Inter-Subunit FRET Experiments.

fMet-$tRNA^{fMet}$ bound 30S pre-initiated complexes (PICs), Cy3B labeled on the 16S rRNA (Marshall et al., 2008), were formed on the ADR1a-SecM(*L*=24; Δ1-158)

mRNA constructs and immobilized to the surface of pre-treated zero-mode waveguide (ZMW) chips (SMRT Cell, *Pacific Biosciences*) through hybridization of the mRNAs to biotinylated splint DNA oligos (Tsai et al., 2014). Elongation mixtures, containing 200 nM fluorescence-quencher-labeled (BHQ-2) 50S ribosomal subunits, 240 nM EF-G, and 3 μM total aa-tRNA·EF-Tu·GTP ternary complex (TC), were delivered to the ZMW chips in a modified PacBio RS sequencer where all individual ZMWs are illuminated with 532 nm laser and fluorescence data is acquired over time (Tsai et al., 2014). Preparation of native or fluorophore-labeled biomolecules was performed as described in (Johansson et al., 2014) and references therein. The elongation reactions were carried out in a Tris-based polymix buffer at 20°C in the presence of 1 μM IF2, 4 mM GTP, 2 mM Trolox and a PCA/PCD oxygen-scavenging system. Fluorescence data was collected at 10 Hz for 10 min, and filtered and analyzed using MATLAB (MathWorks) scripts as has been described previously (Tsai et al., 2014). ZMW chips were loaded stochastically at 30 % occupancy. The 30 % ZMWs with lowest signal were used to calculate background and σ. The wells with signal greater than n*σ above background, that lasted longer than 10 s, were selected for n = N[1 10]. The minimal of discrete differential of the resulting function (number of picked wells from n) was used to identify the best n value for molecule identification. The picked traces were manually curated for fluorescence intensity, fluorescence lifetime, and the fluorescence intensity change when the laser is turned on, to assure single occupancy of ribosomal subunits in the wells. Intersubunit FRET states were assigned based on a hidden Markov model approach (McKinney et al., 2006) with manual correction, from productive traces showing stable [Cy3B]30S immobilization, joining of 50S (yielding high-FRET), and at least two high-low-high-FRET cycles signaling intersubunit rotation (Tsai et al.,

2014). Average state lifetimes were calculated by fitting the individual lifetimes to a single-exponential distribution using maximum-likelihood parameter estimation. Only lifetimes from productive states were included (i.e., low-FRET states that was followed by a high-FRET state and vice versa) to eliminate artifacts from photophysical effects. The density of elongating ribosomes, decreasing with codon number due to both photobleaching and erroneous translation termination, was calculated and summarized from assigned FRET states in n individual traces.

Molecular Dynamics Simulations

The cotranslational folding curve of the ribosome-ADR1a nascent chain complex was calculated on an arrested ribosome using the coarse-grained model of O'Brien and co-workers (O'Brien et al., 2011, 2012) in which amino-acids are represented as one interaction site, purine containing nucleotides as three interaction sites and pyrimidine containing nucleotides as four interaction sites. In this model electrostatic interactions are treated using Debye-Huckel theory, with a 10 Å Debye screening length. We utilized the force-field and Langevin Dynamics protocol published previously (O'Brien et al., 2011, 2012). Briefly, a structure-based force-field (Ueda et al., 1978; Onuchic and Wolynes, 2004) was used for the ADR1a zinc finger domain (PDB ID: 2ADR) and a transferable force field (O'Brien et al., 2011) was used for the unstructured linkers. In the simulations, the 50S subunit of the *E. coli* ribosome (PDB ID: 3UOS) and the nascent chain are explicitly represented. Zinc ions were not represented in the simulation, instead their effect on protein stability was implicitly accounted for by linearly scaling the Lennard-Jones well-depth of residue pairs that are in contact in the native state (O'Brien et al., 2011, 2012) such that the stability of the folded zinc-finger in isolation was equal to -2.0 kcal/mol at 310 K. ADR1a was then covalently attached to unstructured linkers having the same sequences as used in

the experiments (see Fig. S1). Linker lengths of 17 to 46 residues were simulated. At each linker length, replica-exchange simulations (Sugita and Okamoto, 1999) were run with 8 temperature windows ranging between 290 and 370 K. A simulation structure of the zinc finger domain was classified as folded if its root-mean-squared deviation (RMSD) from the ADR1a NMR structure was < 3.5 Å, and classified as unfolded if the RMSD > 5.5 Å. The WHAM equations (Kumar et al., 1992) were then utilized to calculate the probability of the domain being folded as a function of linker length at 310 K.

Cloning and Purification of ADR1a-SecM(*Ms*-Sup1, *L*=25) Ribosome-Nascent Chain Complexes

The ADR1a-SecM(*L*=25) construct, which is at the peak of the force profile in Fig. 1d, was chosen for large-scale preparation and cryo-EM analysis. The *E. coli* SecM stalling sequence was modified by mutating 5 residues to obtain the Sup1 version of the *M. succiniproducens* SecM AP (HPPIRGSP) (Yap and Bernstein, 2009). The resulting sequence was overlapped by PCR to the DNA fragment encoding the last 29 amino acids of the yeast ADR1a protein, yielding ADR1a-SecM(*Ms*-Sup1; *L*=25), Fig. S4a. Primers containing 5′ SapI sites were used to PCR-amplify ADR1a-SecM(*Ms*-Sup1; *L*=25), which was subsequently cloned into a p7XNH vector by using the FX cloning method (Geertsma, 2014). 5 ml of the FX cloning reaction were used to transform chemically competent *E. coli* MC1061 cells. Single colonies were further used to prepare plasmids that were sequence verified and used for *in vitro* translation experiments. The final sequence used was MHHHHHHHHHHLEVLFQGPSYPYDVPDYAKPYPCGLCNR CFTRRDLLIRHAQKIHSGNSGSGVMSSFSTPVWISQHPPIRGSPA, including N-terminal His_{10}-CT-HA tags for purification.

In vitro translation was performed by using the PURE System (NEB) following the manufacturer's instructions. A 500 µl PURE System reaction was incubated for 90 minutes at 37°C, in presence of 50 µM $ZnCl_2$. Ribosomes were then pelleted trough a sucrose-cushion (50 mM Hepes pH 7.2, 250 mM KOAc, 25 mM $Mg[OAc]_2$, 75 mM sucrose) for 1 hour at 100.000 x g at 4 °C. The ribosomal pellet was resuspended in equilibration buffer (50 mM Hepes pH 7.4, 500 mM KOAc, 25 mM $Mg[OAc]_2$, 0.03% DDM, 50 µM $ZnCl_2$, 125 mM sucrose) and incubated with 400 ml Ni-NTA resin (Protino, Macheray-Nagel, Germany) for 1 hour at 4°C. The resin was subsequently washed with ten column volumes (CV) of 50mM Hepes pH 7.4, 500 mM KOAc, 25 mM $Mg[OAc]_2$, 0.03% DDM, 50 µM $ZnCl_2$, 125 mM sucrose, 20 mM imidazole. Ribosome nascent-chain complexes (RNCs) were eluted in 0.5 CV fractions with 50 mM Hepes pH 7.2 , 500 mM KOAc, 25 mM $Mg[OAc]_2$, 0.03% DDM, 50 µM $ZnCl_2$, 125 mM sucrose, 300 mM imidazole. The elution fractions were subsequently loaded onto a 10-40% density sucrose gradient and centrifuged at 45.000 rpm in a SW40 rotor (Beckman Coulter) for three hours. The 70s peak was collected and RNCs were concentrated by ultracentrifugation (100.000xg for 1 hour). The ribosome pellet was finally resuspended in cryo-grid buffer (20 mM Hepes pH 7.2 , 50 mM KOAc, 5 mM $Mg[OAc]_2$, 0.03% DDM, 50 µM $ZnCl_2$, 125 mM sucrose) to a final $O.D._{260}$ = 6, aliquoted in small volumes, and finally stored at -80°C until needed for the preparation of cryo-EM grids. Correct size of the RNC-complexes were verified by 12% Nu-Page (Life Technologies Inc.), and gels were submitted to semidry electroblotting and immunodetection with a primary antibody raised against a HA-tag (Roche). Chemiluminescence detection was done by using the SuperSignal West Dura Extended Duration Substrate (Thermo Scientific) and the Fujifilm LAS-3000 imaging system.

Cryo-EM Specimen Preparation, Data Collection, Processing, and Model Building

Carbon-coated holey grid preparation of ADR1a-SecM(*Ms*-Sup1; *L*=25) RNCs was carried out as described previously (Bischoff et al., 2014). Cryo-EM data was collected on a Titan Krios TEM (FEI, USA) operated at 300 keV and equipped with a back-thinned Falcon II (FEI, USA) direct electron detector. The camera was calibrated for a nominal magnification of 75,000x, resulting in a pixel size of 1.37 Å at the specimen. Seven blocks of frames s^{-1} were recorded in automatic mode with a dose of 5 $e^{-}/Å^{2}$ per block at defocus values between -1 and -3.2 mm. Frames were aligned using the software developed by the Yifang Cheng lab at UCSF (Li et al., 2013).

Micrographs showing drift or contamination were manually discarded from the dataset. All processing was performed using the SPIDER software package (Frank et al., 1996). The initial dataset of 496,340 particles was first cleaned from non-ribosomal particles (306,243 ribosomal particles left) and subsequently sorted for the presence of A, P and E site tRNAs. The dataset that contained strong density for tRNA in the P-site, was further refined by applying a cross correlation cut-off. The final dataset contained 151,900 particles and was refined to a final average resolution of 4.8 Å according to the FSC criterion at cut-off at 0.14. Potential over-fitting was excluded by truncating high frequencies (low-pass filter at 8 Å) during the whole refinement process (Scheres and Chen, 2012).

For structural comparison and interpretation of the cryo-EM density obtained, we fitted the structure of the *E. coli* 70S ribosome (PDB ID: 3OFR), using UCSF Chimera (Pettersen et al., 2004). A poly-alanine model of the SecM-stalled nascent chain was built based on the model of a TnaC stalled peptide (PDB ID: 4YU8)

(Bischoff et al., 2014). The extra density at the end of the stalled SecM was extracted and compared with PDB-derived density maps of the ADR1a domain (PDB ID: 2ADR) at different resolutions and contour levels. Finally, the structure of the ADR1a domain was rigid-body fitted according to the highest cross-correlation between the density model maps and the electron density.

SUPPLEMENTAL REFERENCES

Bischoff, L., Berninghausen, O., and Beckmann, R. (2014). Molecular basis for the ribosome functioning as an L-tryptophan sensor. Cell reports *9*, 469-475.

Frank, J., Radermacher, M., Penczek, P., Zhu, J., Li, Y., Ladjadj, M., and Leith, A. (1996). SPIDER and WEB: processing and visualization of images in 3D electron microscopy and related fields. Journal of structural biology *116*, 190-199.

Geertsma, E.R. (2014). FX cloning: a simple and robust high-throughput cloning method for protein expression. Methods Mol Biol *1116*, 153-164.

Ismail, N., Hedman, R., Schiller, N., and von Heijne, G. (2012). A biphasic pulling force acts on transmembrane helices during translocon-mediated membrane integration. Nature Struct Molec Biol *19*, 1018-1022.

Johansson, M., Chen, J., Tsai, A., Kornberg, G., and Puglisi, J.D. (2014). Sequence-dependent elongation dynamics on macrolide-bound ribosomes. Cell reports *7*, 1534-1546.

Kumar, S., Rosenberg, J.M., Bouzida, D., Swendsen, R.H., and Kollman, P.A. (1992). THE weighted histogram analysis method for free-energy calculations on biomolecules. I. The method. J Comput Chem *13*, 1011-1021.

Li, X., Mooney, P., Zheng, S., Booth, C.R., Braunfeld, M.B., Gubbens, S., Agard, D.A., and Cheng, Y. (2013). Electron counting and beam-induced motion

correction enable near-atomic-resolution single-particle cryo-EM. Nat Methods *10*, 584-590.

Marshall, R.A., Dorywalska, M., and Puglisi, J.D. (2008). Irreversible chemical steps control intersubunit dynamics during translation. Proc Natl Acad Sci U S A *105*, 15364-15369.

McKinney, S.A., Joo, C., and Ha, T. (2006). Analysis of single-molecule FRET trajectories using hidden Markov modeling. Biophys J *91*, 1941-1951.

O'Brien, E.P., Christodoulou, J., Vendruscolo, M., and Dobson, C.M. (2011). New scenarios of protein folding can occur on the ribosome. J Am Chem Soc *133*, 513-526.

O'Brien, E.P., Christodoulou, J., Vendruscolo, M., and Dobson, C.M. (2012). Trigger factor slows co-translational folding through kinetic trapping while sterically protecting the nascent chain from aberrant cytosolic interactions. J Am Chem Soc *134*, 10920-10932.

Onuchic, J.N., and Wolynes, P.G. (2004). Theory of protein folding. Curr Opin Struct Biol *14*, 70-75.

Pettersen, E.F., Goddard, T.D., Huang, C.C., Couch, G.S., Greenblatt, D.M., Meng, E.C., and Ferrin, T.E. (2004). UCSF Chimera--a visualization system for exploratory research and analysis. Journal of computational chemistry *25*, 1605-1612.

Scheres, S.H., and Chen, S. (2012). Prevention of overfitting in cryo-EM structure determination. Nat Methods *9*, 853-854.

Schwarz, D., Junge, F., Durst, F., Frölich, N., Schneider, B., Reckel, S., Sobhanifar, S., Dötsch, V., and Bernhard, F. (2007). Preparative scale expression of

membrane proteins in Escherichia coli-based continuous exchange cell-free systems. Nat Protoc *2*, 2945-2957.

Shimizu, Y., Kanamori, T., and Ueda, T. (2005). Protein synthesis by pure translation systems. Methods *36*, 299-304.

Sugita, Y., and Okamoto, Y. (1999). Replica-exchange molecular dynamics method for protein folding. Chem Phys Lett *314*, 141-151.

Tsai, A., Kornberg, G., Johansson, M., Chen, J., and Puglisi, J.D. (2014). The dynamics of SecM-induced translational stalling. Cell reports *7*, 1521-1533.

Ueda, Y., Taketomi, H., and Gō, N. (1978). Studies on protein folding, unfolding, and fluctuations by computer simulation. II. A. Three-dimensional lattice model of lysozyme. Biopolymers *17*, 1531–1548.

Welte, T., Kudva, R., Kuhn, P., Sturm, L., Braig, D., Muller, M., Warscheid, B., Drepper, F., and Koch, H.G. (2012). Promiscuous targeting of polytopic membrane proteins to SecYEG or YidC by the Escherichia coli signal recognition particle. Mol Biol Cell *23*, 464-479.

Yap, M.N., and Bernstein, H.D. (2009). The plasticity of a translation arrest motif yields insights into nascent polypeptide recognition inside the ribosome tunnel. Mol Cell *34*, 201-211.
