# Supplementary material for: Cotranslational Protein Folding inside the Ribosome Exit Tunnel
Source: Cell Rep. 2015 Aug 28;12(10):1533–40. doi: 10.1016/j.celrep.2015.07.065 (PMC4571824; doi:10.1016/j.celrep.2015.07.065)
Supplement: Document S2. Article plus Supplemental Information [file mmc2.pdf]

Cell Reports

Report

Cotranslational Protein Folding inside the Ribosome Exit Tunnel

Graphical Abstract

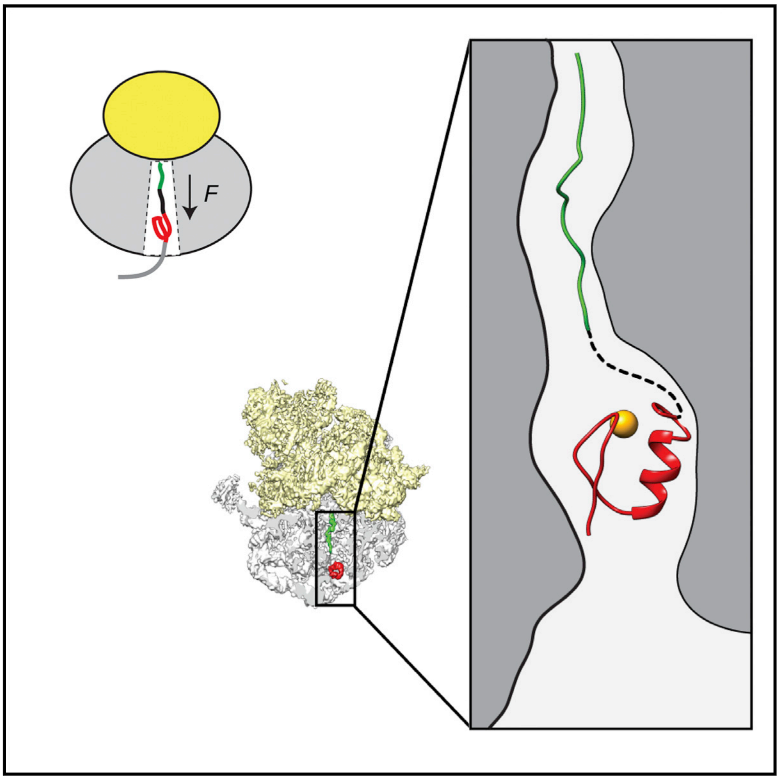

Highlights

- Cotranslational folding is studied by arrest-peptide-mediated force measurements
- Single-molecule measurements show that a pulling force prevents ribosome stalling
- A ribosome-tethered zinc-finger domain is visualized by cryo-EM (electron microscopy)
- The zinc-finger domain is shown to fold deep inside the ribosome exit tunnel

Authors

Ola B. Nilsson, Rickard Hedman, Jacopo Marino, ..., Edward P. O'Brien, Roland Beckmann, Gunnar von Heijne

Correspondence

gunnar@dbb.su.se

In Brief

Nilsson et al. present an integrated approach to the study of cotranslational protein folding, in which the folding transition is mapped by arrest-peptide-mediated force measurements, molecular dynamics simulations, and cryo-EM (electron microscopy). The small zinc-finger domain ADR1a is shown to fold deep inside the ribosome exit tunnel.

Nilsson et al., 2015, Cell Reports *12*, 1533–1540
September 8, 2015 ©2015 The Authors
http://dx.doi.org/10.1016/j.celrep.2015.07.065

Cell Reports
Report

OPEN ACCESS
CellPress

Cotranslational Protein Folding inside the Ribosome Exit Tunnel

Ola B. Nilsson,[1,8] Rickard Hedman,[1,8] Jacopo Marino,[2] Stephan Wickles,[2] Lukas Bischoff,[2] Magnus Johansson,[3] Annika Müller-Lucks,[1] Fabio Trovato,[6] Joseph D. Puglisi,[4,5] Edward P. O'Brien,[6] Roland Beckmann,[2] and Gunnar von Heijne[1,7,*]

[1]Department of Biochemistry and Biophysics, Center for Biomembrane Research, Stockholm University, 106 91 Stockholm, Sweden
[2]Gene Center and Center for Integrated Protein Science Munich, CiPS-M, Feodor-Lynen-Strasse 25, University of Munich, 81377 Munich, Germany
[3]Department of Cell and Molecular Biology, Biomedical Center, Uppsala University, Box 596, 751 24 Uppsala, Sweden
[4]Department of Structural Biology
[5]Stanford Magnetic Resonance Laboratory
Stanford University School of Medicine, Stanford, CA 94305-5126, USA
[6]Department of Chemistry, Pennsylvania State University, University Park, PA 16802, USA
[7]Science for Life Laboratory, Stockholm University, Box 1031, 171 21 Solna, Sweden
[8]Co-first author
*Correspondence: gunnar@dbb.su.se
http://dx.doi.org/10.1016/j.celrep.2015.07.065
This is an open access article under the CC BY license (http://creativecommons.org/licenses/by/4.0/).

SUMMARY

At what point during translation do proteins fold? It is well established that proteins can fold cotranslationally outside the ribosome exit tunnel, whereas studies of folding inside the exit tunnel have so far detected only the formation of helical secondary structure and collapsed or partially structured folding intermediates. Here, using a combination of cotranslational nascent chain force measurements, inter-subunit fluorescence resonance energy transfer studies on single translating ribosomes, molecular dynamics simulations, and cryoelectron microscopy, we show that a small zinc-finger domain protein can fold deep inside the vestibule of the ribosome exit tunnel. Thus, for small protein domains, the ribosome itself can provide the kind of sheltered folding environment that chaperones provide for larger proteins.

INTRODUCTION

Cotranslational folding of proteins that have emerged from the ribosome exit tunnel has been studied for decades using either stalled ribosome-nascent chain complexes (RNCs) (Kolb et al., 2000; Kowarik et al., 2002; Kosolapov and Deutsch, 2009; Kaiser et al., 2011; Kelkar et al., 2012; Lin et al., 2012; Waudby et al., 2013; Nissley and O'Brien, 2014) or kinetic measurements (Nicola et al., 1999). Isolated elements of secondary structure and collapsed or partially structured folding intermediates have been detected inside the exit tunnel (Mingarro et al., 2000; Bhushan et al., 2010; Tu et al., 2014), but in no case has a protein domain been shown to be able to fold into its native structure while still inside the ribosome.

On the basis of our finding that cotranslational processes such as protein translocation across, or insertion into, a membrane generate pulling forces on the nascent polypeptide chain (Ismail et al., 2012, 2015; Cymer and von Heijne, 2013), we previously suggested that proteins that start to fold cotranslationally while still in contact with the ribosome should exert a similar pulling force on the nascent chain (Ismail et al., 2012); that is, the free energy released by the folding reaction should be at least in part stored as an increased tension in the nascent chain (Figure 1A). This was recently confirmed in a study of cotranslational folding of the Top7 protein, a 93-residue protein that folds just outside the exit tunnel while exerting a force of ~10 pN on the nascent chain (Goldman et al., 2015).

Here, we have set out to follow the folding of a protein domain as it progressively moves out of the ribosome in step with chain elongation, using an assay that takes advantage of the force sensitivity of translational arrest peptides (APs) (Butkus et al., 2003; Ismail et al., 2012; Goldman et al., 2015). APs from bacterial SecM proteins are exquisitely sensitive to the tension present in the nascent chain at the precise moment when the ribosome translates the last codon in the AP, with the efficiency of the translational arrest being reduced in proportion to an increase in tension (Ismail et al., 2012, 2015; Goldman et al., 2015). By making a series of constructs in which a suitable AP is separated by a varying number of residues, *L*, from the protein to be studied and measuring the efficiency of translational arrest for each value of *L*, we can obtain an indirect measure of the instantaneous tension in the nascent chain during translation. We now demonstrate that such "force profiles" appear to give a direct insight into the folding transition that a protein undergoes as it passes down the exit tunnel. We compare the experimental profile recorded for a small zinc-finger domain with folding simulations based on coarse-grained molecular dynamics and determine the location of the domain inside the ribosome exit tunnel at the point of maximal folding force by cryo-EM (electron microscopy). Our results show that small protein domains can fold

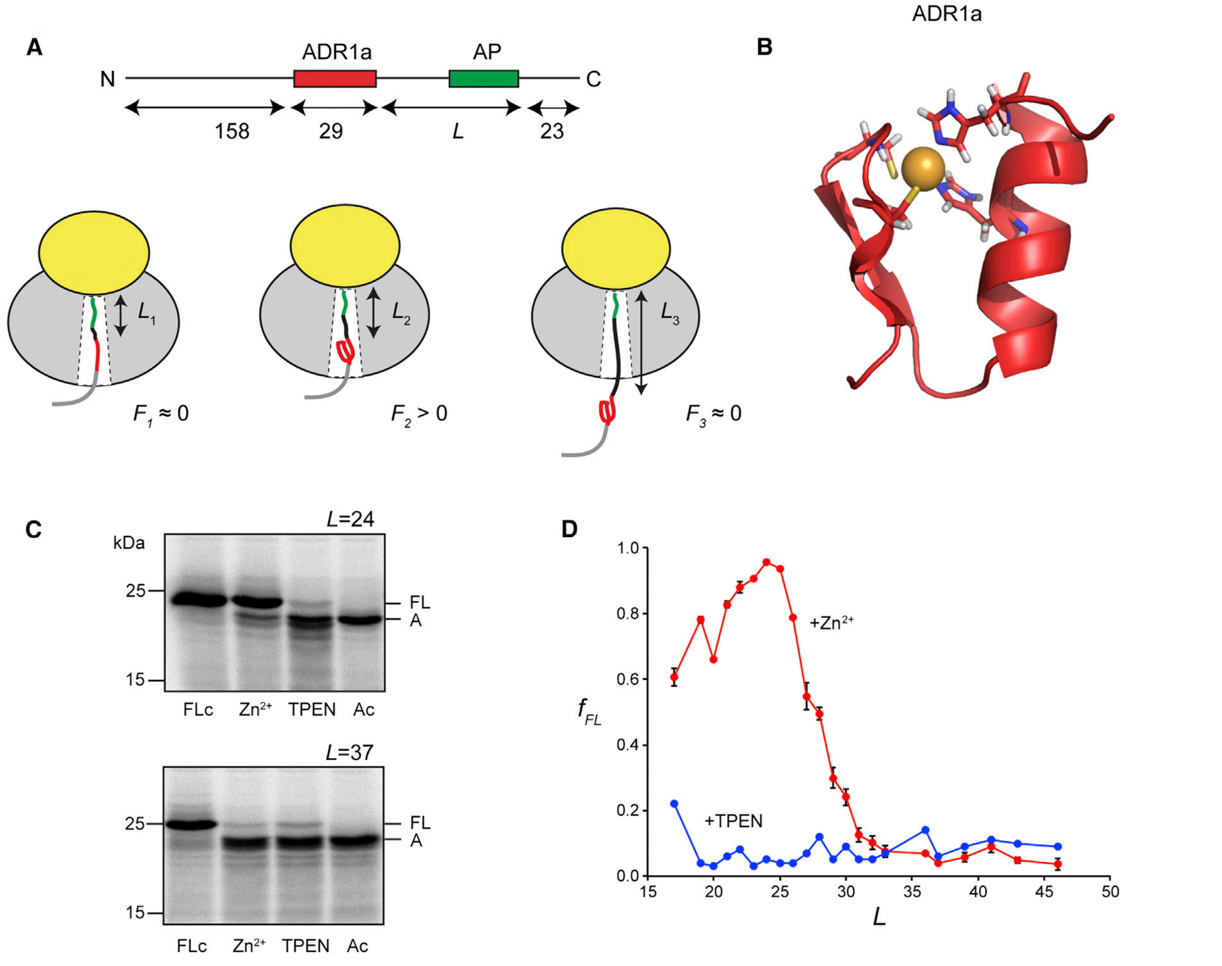

Figure 1. Cotranslational Folding of the ADR1a Zinc-Finger Domain

(A) Force measurement assay. The ADR1a domain is placed *L* residues away from the C-terminal Pro residue in the *E. coli* SecM AP. An unrelated segment from the *E. coli* LepB protein (LepB residues 78–226) is added to the N terminus in order to increase the size of the protein such that it can be readily visualized by SDS-PAGE, and a 23-residue C-terminal segment ensures that arrested and full-length forms of the protein can be easily separated on the gel. The LepB part is composed of five small β-hairpin segments that do not interact with one another in the LepB structure (PDB: 1B12) and hence cannot fold in itself. The cartoon below shows three ADR1a-AP constructs with different values of *L* ($L_1 < L_2 < L_3$). The ribosomal tunnel is too tight for the protein to fold at L_1, and the protein is already folded and outside the tunnel when the ribosome reasches the AP at L_3. Only at L_2 will folding of the protein against the widening ribosomal exit tunnel generate a pulling force *F* on the AP, leading to inefficient ribosomal stalling and an increase in the fraction full-length protein, f_{FL}.

(B) Structure of ADR1a (PDB: 2ADR). The Zn^{2+} ion is shown in gold.

(C) In vitro translation the PURE system of the ADR1a-SecM (*L* = 24) (top) and ADR1a-SecM (*L* = 37) (bottom) constructs. Full-length (FL) and arrested (A) forms are indicated. Ac, control construct with a stop codon inserted directly after the AP; FLc, full-length control construct, where the critical Pro at the end of the AP is mutated to Ala; TPEN, translation carried out in the presence of 50 μM of the Zn^{2+} chelator TPEN; Zn^{2+}, translation carried out in the presence of 50 μM Zn^{2+}.

(D) Fraction full-length protein, f_{FL}, plotted as a function of *L* for the ADR1a-AP constructs translated in the PURE in vitro system either in the absence (blue curve; to deplete the translation mix of Zn^{2+}, the Zn^{2+} chelator TPEN was included at 50 μM) or presence (red curve) of 50 μM Zn^{2+}. SEMs are indicated.

See also Figures S1 and S2.

while still in the exit tunnel and establish the use of AP-mediated force measurements for the study of cotranslational protein folding.

RESULTS

To explore the possibility of protein folding inside the exit tunnel, we chose the second of the two zinc-finger domains in the yeast ADR1 protein (Párraga et al., 1988) (Figure 1B). The domain (called ADR1a) is 29 residues long and folds around a Zn^{2+} ion using two histidines and two cysteines to chelate the ion. The folding of ADR1a is totally dependent on the presence of Zn^{2+} (Párraga et al., 1988) and can hence be easily manipulated (Conti et al., 2014). Moreover, the protein is small enough that it might be able to fold inside the exit tunnel, a possibility suggested by previous theoretical studies (O'Brien et al., 2010, 2011).

We made a series of constructs in which ADR1a is placed *L* residues upstream of the critical C-terminal Pro residue in the

relatively weakly stalling *Escherichia coli* SecM AP (Yap and Bernstein, 2009; Ismail et al., 2012; Goldman et al., 2015), which in turn is 23 residues upstream of the stop codon (Figure 1A; see Figure S1 for amino acid sequences). In constructs in which there is little tension in the nascent chain at the precise moment when the ribosome reaches the critical Pro codon, the ribosome will stall on the AP and a short, arrested version of the protein will be produced. In contrast, in constructs in which there is high tension (~10 pN or more; Goldman et al., 2015) in the nascent chain at this moment, stalling will be inefficient, and mostly full-length protein will be produced. The fraction full-length protein, f_{FL}, can therefore serve as a proxy for the tension in the nascent chain, as shown in previous studies (Ismail et al., 2012, 2015).

ADR1a Folds inside the Ribosome Exit Tunnel

Translation of ADR1a-SecM constructs in the PURE in vitro translation system (Shimizu et al., 2001, 2005), either in a Zn^{2+}-depleted translation mix or in the presence of 50 μM Zn^{2+} (Figures 1C and 1D), showed efficient stalling in the absence of Zn^{2+} ($f_{FL} \approx 0.1$ for all values of L). In the presence of Zn^{2+}, the picture is dramatically different, with f_{FL} starting to increase at $L \approx 20$ residues, going through a sharp maximum at L_{max} = 24–26 residues and returning to baseline at $L \approx 30$ residues. Mutating one or both of the Zn^{2+}-binding His residues in ADR1a-SecM (L = 24) to Ala returns f_{FL} to baseline (Figure S2A). Translation of ADR1a-SecM constructs in an *E. coli* S135 extract yields similar results, albeit with a lower maximal value of f_{FL} (Figure S2B). Titration of Zn^{2+} in the S135 extract translation reaction shows that the half-maximal f_{FL} value is reached at $[Zn^{2+}] \approx 1$ μM (Figure S2C); Zn^{2+} dissociation constants for typical zinc-finger domains are in the range 0.1–2 μM (Rich et al., 2012). A small signal can also be detected at L_{max} = 25–27 residues when the constructs are expressed in live *E. coli* cells in the absence or presence of 500 μM Zn^{2+} in the medium (Figure S2B). We conclude that ribosomal stalling on the SecM AP is prevented when ADR1a folds and that, as it takes about 30 residues of extended nascent chain to span the ~100 Å from the P-site to the tunnel exit (Bhushan et al., 2011), ADR1a folds inside the exit tunnel. This conclusion holds regardless of whether translation is carried out in vitro or in vivo.

Analysis of Ribosome Stalling on the SecM AP by Single-Ribosome Tracking

To characterize the effect of protein folding on the AP-induced translational arrest in more detail, we applied real-time fluorescence resonance energy transfer (FRET)-based single-ribosome tracking of ribosomes translating the ADR1a-SecM (L = 24) construct, that is, a construct for which there is very little stalling at 50 μM Zn^{2+} (see Figure 1C). In this case, the N-terminal 158-residue-long segment upstream of ADR1a was deleted (compare Figures 1A and S1). Using a previously established method of attaching fluorescent probes to the large and small ribosomal subunits, the transitions between the non-rotated and rotated states of individual ribosomes can be tracked as they translate along an mRNA (Marshall et al., 2008) (Figures 2A and S3), providing translation times at each codon. As shown in Figure 2B, very few ribosomes translate beyond the AP when translation of the ADR1a-SecM (L = 24) construct is carried out in the absence of Zn^{2+}, as has been shown previously for another SecM construct (Tsai et al., 2014). Mutating the critical Pro residue at the end of the AP to Ala inhibits stalling, as expected (Figure 2C). Strikingly, when translation of the ADR1a-SecM (L = 24) construct is carried out in the presence of 50 μM Zn^{2+}, stalling is completely inhibited, and ribosomes progress unhindered beyond the AP (Figure 2D), providing additional evidence of a SecM AP response to the folding of ADR1a. The complete disappearance of the stalling signal in the presence of Zn^{2+} (i.e., no long dwells in the rotated state beyond codon 48 or in the non-rotated state at codons around Pro54, compare Figures 2B and 2D) suggests that folding of the ADR1a domain occurs on a significantly shorter timescale than the elongation timescale in the experimental setup (~5 s per codon) and that the pulling force exerted by the ADR1a domain is present already when the ribosome is a few codons upstream of the critical Pro54 codon, in accordance with the data shown in Figure 1D. It should be noted that the mRNA construct in the present study is much longer than what has been used before in these types of experiment (Tsai et al., 2014) and that uncertainty in codon assignment increases with codon number because of possible mis-assignments of state transitions (e.g., because of fluorophore blinking or very fast state transitions). However, an uncertainty in exact codon numbering around the arrest codons does not affect our conclusions.

Molecular Dynamics Modeling of ADR1a Folding in the Exit Tunnel

We next used a previously developed coarse-grained molecular dynamics protocol (O'Brien et al., 2010) to ask whether, as suggested by the f_{FL} profile measurements, there is enough room in the exit tunnel to allow folding of ADR1a. ADR1a-SecM constructs with different tether lengths were modeled into a high-resolution structure of the *E. coli* ribosome (Zhou et al., 2012), and replica-exchange Langevin dynamics (Sugita and Okamoto, 1999) were run at each tether length. Figure 3A shows the probability that the ADR1a segment is found in its folded state as a function of tether length. The folding transition is predicted to take place over the interval $L \approx 24$–32 residues with a midpoint at L = 28 residues (i.e., at ~3 residues higher L values than seen in the force profile). Analysis of the data obtained with a tether length of L = 25 residues shows that folded ADR1a is found in the exit tunnel, with His_{21} located at a distance of 65–75 Å from the tRNA (Figure 3B, top). To provide a comparison with the cryo-EM results reported below, a simulation of the L = 25 construct was also carried out at T = 140 K, just above the water glass transition temperature; under these conditions, folded ADR1a is found in a more restricted portion of the exit tunnel, with His_{21} located 65–67 Å from the tRNA (Figure 3B, bottom).

Visualization of ADR1a in the Exit Tunnel by Cryo-EM

To confirm that ADR1a folds inside the exit tunnel, we sought to visualize it in the ribosome at tether length $L = L_{max}$ (i.e., at the top of the f_{FL} profile; Figure 1D) by cryo-EM. Because, with the relatively weak SecM AP from *E. coli*, $f_{FL} \approx 1$ at $L = L_{max}$, stably stalled ribosome-ADR1a-SecM complexes of this kind cannot be isolated. We therefore introduced the previously described strongly stalling SecM (*Ms*-Sup1) AP (Ismail et al., 2012) instead (Figure S4A). Using the ADR1a-SecM

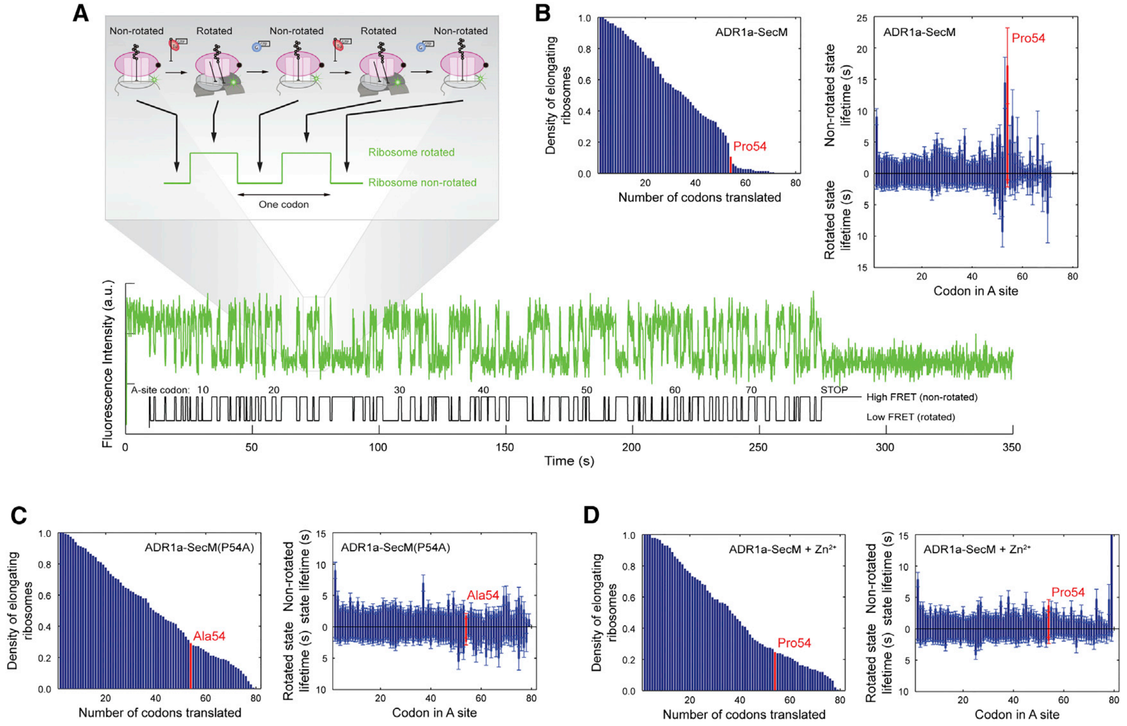

Figure 2. Folding of the ADR1a Domain Prevents Ribosomal Stalling on the SecM AP

(A) The dynamics of ADR1a-SecM (*L* = 24; Δ1–158) translation were assayed in real time using inter-subunit FRET between Cy3B and BHQ-2. Transition from low Cy3B intensity (high FRET) to high intensity (low FRET) and back to low intensity again, as a consequence of the inter-subunit rotations, reports on one elongation cycle (i.e., peptidyl transfer and subsequent translocation). The time-trace example shows how one ribosome translates the whole ADR1a-SecM (*L* = 24) ORF in a construct in which the critical C-terminal Pro54 in the AP has been changed to Ala.

(B) Survival plot (left) and lifetimes of the rotated and non-rotated states (right) for each individual codon summarized from ADR1a-SecM (*L* = 24) translation time traces (n = 149). The critical Pro54 codon is shown in red for clarity.

(C) Inter-subunit FRET data from elongation of an ADR1a-SecM (*L* = 24) construct in which the Pro54 has been changed to Ala (n = 147).

(D) Inter-subunit FRET data from elongation of the ADR1a-SecM (*L* = 24) construct in the presence of 50 μM Zn^{2+} (n = 147). Lifetimes are fitted to single-exponential distributions. SEMs are indicated.

See also Figure S3.

(*Ms*-Sup1; *L* = 25) construct (with an added N-terminal purification tag and lacking the 158-residue-long segment upstream of ADR1a; compare Figure 1A), we purified stalled RNCs from a translation reaction in the PURE system supplemented with 50 μM Zn^{2+} (Figures S4B and S4C) and obtained a 4.8 Å 3D reconstruction by cryo-EM (Figures 4 and S4D–S4F). Parts of the SecM AP can be seen in the exit tunnel, as well as the P-site tRNA. Strikingly, an extra density, not present in empty ribosomes, is clearly visible in the exit tunnel, ~60 Å from the tRNA (Figure 4A). Rigid-body docking of a molecular model of ADR1a derived from nuclear magnetic resonance (NMR) analysis (Protein Data Bank [PDB]: 2ADR) revealed an excellent fit, with a cross-correlation of 0.93 between the model and the density. The ADR1a domain is lodged between ribosomal proteins uL22 and uL23 and ribosomal rRNA helices H23, H24, and H50 (Figure 4C) and is located a few angstroms deeper in the exit tunnel in the cryo-EM reconstruction than in the ensemble of folded structures seen in the 140 K molecular dynamics trajectory (Figure 3B, bottom, arrow). The ADR1a snapshot structure (gold) from the 140 K simulation ensemble that best fits the cryo-EM reconstruction (red) in the exit tunnel is shown in Figure 3C.

DISCUSSION

Here, we present an integrated approach to the study of cotranslational protein folding, in which the folding transition as a function of position relative to the exit tunnel is mapped by AP-mediated force measurements and molecular dynamics simulations. The location of the partially or fully folded protein or protein domain in the ribosome at relevant *L* values is then determined by mutating the AP such that it can withstand the folding force (see Cymer et al., 2015, for a large collection of APs of different stalling potency) and determining the structure of the resulting RNCs by cryo-EM. Using this approach, we show that the small zinc-finger domain ADR1a folds cotranslationally as the tether connecting it to the ribosome grows in length from ~20 to ~30 residues. Both coarse-grained molecular dynamics simulation and cryo-EM visualization of ribosome-bound ADR1a at a tether length corresponding to the midpoint of the folding transition show ADR1a buried deep in the vestibule of the exit tunnel, providing a clear demonstration that small proteins or protein domains can fold within the ribosome, as predicted by computational studies (O'Brien et al., 2010, 2011). Although the zinc finger

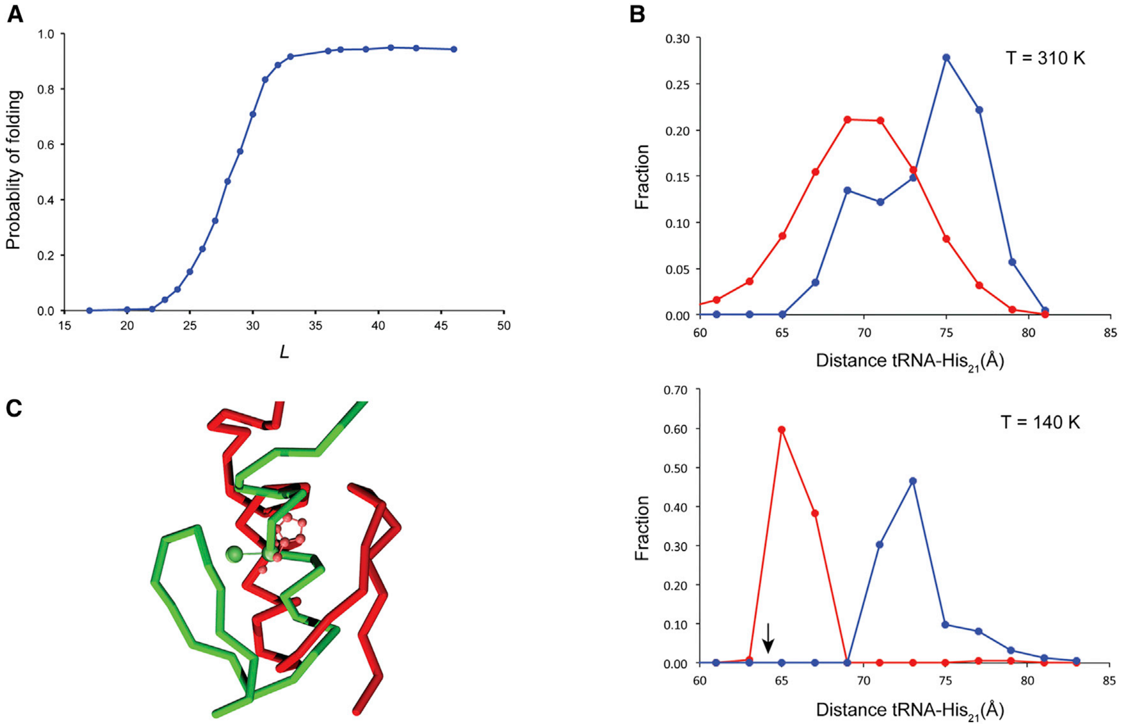

Figure 3. Molecular Dynamics Simulation of Cotranslational Folding of ADR1a

(A) The probability that the ADR1a domain is folded at 37°C is plotted as a function of L.

(B) Distance distribution of the folded (root-mean-square deviation [rmsd] < 3.5 Å from the cryo-EM ADR1a model, after alignment of the isolated domains; red) and unfolded (rmsd > 5.5 Å from the cryo-EM ADR1a model; blue) ADR1a domains in the exit tunnel. Distance distributions were calculated as a function of the distance between the last P atom in the tRNA of the cryo-EM structure and the C_{α} of His_{21} in ADR1a (2Å bins). Alignment of the cryo-EM and simulated ribosome structures was performed in advance. Top: Simulation run at 310 K. Bottom: Simulation run at 140 K. The arrow indicates the distance between the tRNA and His_{21} in the cryo-EM reconstruction.

(C) Snapshot of the folded structure of ADR1a (green) from the 140 K simulation that best overlaps the cryo-EM structure (red) in the exit tunnel at tether length L = 25. His_{21} is displayed in in its coarse-grained two-ball representation for the simulation model and in ball-and-stick representation for the cryo-EM structure.

is one of the smallest independently folding protein domains, it has been estimated that ~9% of all structural domains found in the PDB are less than 40 residues long, and ~18% are less than 60 residues long (Wheelan et al., 2000). Folding of protein domains wholly or partly inside the exit tunnel may thus be not too uncommon, despite its relatively constrained geometry (Voss et al., 2006).

Although we cannot completely rule out that ADR1a relieves the translational stall not by exerting a pulling force but by some kind of indirect mechanism whereby, for example, interactions between folded ADR1a and the tunnel wall give rise to a long-range (>60 Å) allosteric effect on the peptidyl transferase center, we consider this unlikely. First, published 3.5–5.5 Å resolution cryo-EM structures of SecM and MifM APs stalled in the exit tunnel show conformational changes in the ribosome only close to the peptidyl transferase center (Bhushan et al., 2011; Sohmen et al., 2015), and not over such long distances as would be required for an allosteric effect of ADR1a. Second, direct pulling on a stalled SecM AP by optical tweezers shows that the mean life time of the stalled state is reduced in proportion to the pulling force (Goldman et al., 2015). Third, qualitatively similar effects on f_{FL} as seen with ADR1a are seen when pulling forces are induced by processes as diverse as the insertion of a transmembrane helix into the inner membrane (Ismail et al., 2012), the translocation of negatively charged residues across the inner membrane (Ismail et al., 2015), and folding of a larger protein, Top7, just outside the exit tunnel (Goldman et al., 2015). The most parsimonious hypothesis is thus that, also for ADR1a, it is the pulling force rather than some ADR1a-specific allosteric interaction with the tunnel wall that is responsible for the variation in f_{FL} with L.

Taken together with a recent study of the Top7 protein (Goldman et al., 2015), our results demonstrate that APs can be used to study folding both inside and outside the exit tunnel; optical tweezer measurements (Goldman et al., 2015) or comparison of AP-based f_{FL} measurements with forces calculated from a physical model of the same process (Ismail et al., 2015) can provide estimates of the relation between the actual folding force (in piconewtons) and f_{FL}. Future studies will allow more precise definitions of how the size and shape of a protein domain dictate where it folds in relation to the exit tunnel and may allow us to probe the interactions between a cotranslationally folding protein and, for example, chaperones or cofactors of various kinds as a function of its degree of exposure outside the ribosome.

OPEN
ACCESS
CellPress

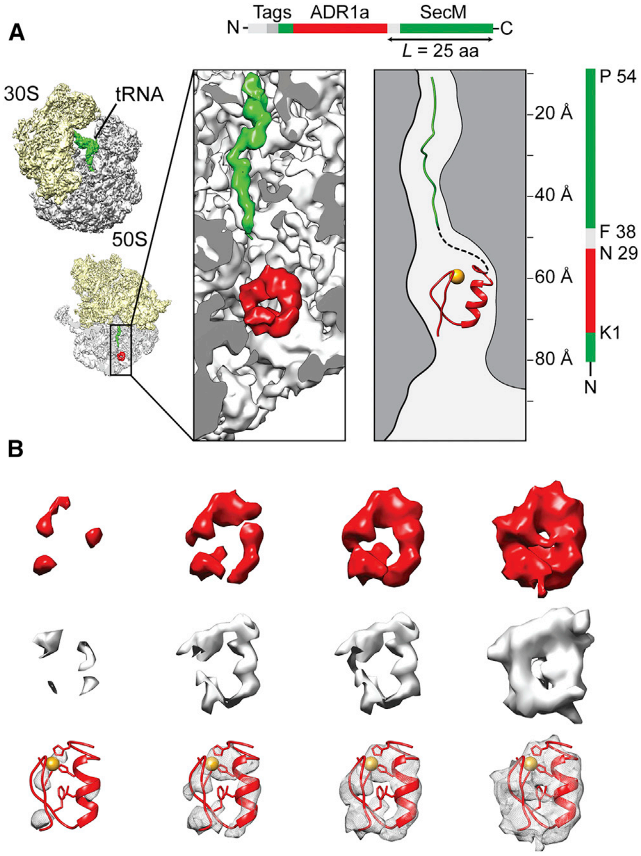

Figure 4. Visualization by Cryo-EM of the ADR1a Domain in a Stalled Ribosome-ADR1a-SecM (*Ms*-Sup1; *L* = 25) Complex

(A) Schematic of the construct used for in vitro translation (top) and cryo-EM reconstructions of stalled *E. coli* ribosome-SecM-ADR1a complexes (left). The 30S subunit is depicted in yellow, the 50S subunit in gray, and the peptidyl-tRNA with the nascent polypeptide chain in green. Additionally, a cross-section through the cryo-EM density is shown in which the density for the nascent chain and the ADR1a domain (PDB: 2ADR) are depicted in green and red, respectively. A close-up of the tunnel and a schematic view are shown with the structure of the ADR1a domain fitted as rigid body depicted in red.

(B) Isolated density for the ADR1a domain (red) shown at different contour levels (top) compared with corresponding densities calculated from the NMR-derived molecular model of ADR1a (middle). Isolated cryo-EM density is shown transparent with the docked model (red) and the coordinated Zn^{2+} ion in yellow (bottom).

See also Figure S4.

EXPERIMENTAL PROCEDURES

Plasmids

All ADR1a constructs were generated from the previously described pING1 plasmid carrying a truncated *lepB* gene containing a [6L,13A] H segment insert and the *E. coli* SecM AP under the control of an arabinose-inducible promoter (Ismail et al., 2012), as detailed in the Supplemental Information. For RNA transcription using the T7 promoter, all constructs were subcloned into plasmid pET19b (Novagen) using *NcoI* and BamHI.

In Vitro Transcription and Translation

In vitro transcription was performed with T7 RNA polymerase according to the manufacturer's protocol (Promega) using PCR products as templates for the generation of truncated nascent chains. RNA obtained was purified using RNeasy Mini Kit (Qiagen). Translation was performed in the commercially available PUREfrex system (Shimizu et al., 2005) and in a Zn^{2+}-free S135 *E. coli* extract (Welte et al., 2012) modified from Schwarz et al. (2007). Proteins were separated by SDS-PAGE, visualized on a Fuji FLA-3000 phosphoimager, and quantified. Values of f_{FL} were calculated as $f_{FL} = I_{FL}/(I_{FL} + I_A)$, where I_{FL} is the intensity of the band corresponding to the full-length protein, and I_A is the intensity of the band corresponding to the arrested form of the protein (compare Figure 1C). Experiments were repeated three times, and SEMs were calculated.

In Vivo Pulse-Labeling Analysis

Expression of ADR1a-SecM constructs in *E. coli* MC1061 cells was induced with arabinose for 5 min. $ZnCl_2$ was added to a final concentration of 0.5 mM at the point of induction. Cells were then pulse-labeled with $[^{35}S]$-Met for 2 min at 37°C, trichloroacetic acid-precipitated, and prepared for SDS-PAGE analysis.

Single-Ribosome Inter-Subunit FRET Experiments

fMet-$tRNA^{fMet}$-bound 30S pre-initiated complexes, Cy3B labeled on the 16S rRNA (Marshall et al., 2008), were formed on the ADR1a-SecM (*L* = 24; Δ1–158) mRNA constructs and immobilized to the surface of pre-treated zero-mode waveguide (ZMW) chips through hybridization of the mRNAs to biotinylated splint DNA oligos (Tsai et al., 2014). Elongation mixtures were delivered to the ZMW chips in a modified PacBio RS sequencer whereby all individual ZMWs are illuminated with 532 nm laser and fluorescence data are acquired over time (Tsai et al., 2014). Preparation of native or fluorophore-labeled biomolecules was performed as described in Johansson et al. (2014). The elongation reactions were carried out in a Tris-based polymix buffer at 20°C in the presence of 1 μM IF2, 4 mM guanosine triphosphate, 2 mM Trolox, and a protocatechuic acid/protocatechuate-3,4-dioxygenase oxygen-scavenging system. Fluorescence data were collected at 10 Hz for 10 min and filtered and analyzed using MATLAB (The MathWorks) scripts, as has been described previously (Tsai et al., 2014).

Molecular Dynamics Simulations

The cotranslational folding curve of the ribosome-ADR1a nascent chain complex was calculated on an arrested ribosome using the coarse-grained model of O'Brien et al (2011, 2012). In the simulations, the 50S subunit of the *E. coli* ribosome (PDB: 3UOS) and the nascent chain are explicitly represented. Zinc ions were not represented in the simulation; instead their effect on protein stability was implicitly accounted for by linearly scaling the Lennard-Jones well depth of residue pairs that are in contact in the native state (O'Brien et al., 2011, 2012) such that the stability of the folded zinc finger in isolation was equal to −2.0 kcal/mol at 310 K. ADR1a was then covalently attached to unstructured linkers having the same sequences as used in the experiments (see Figure S1). At each linker length, replica-exchange simulations (Sugita and Okamoto, 1999) were run with eight temperature windows ranging between 290 and 370 K.

Cloning and Purification of ADR1a-SecM (*Ms*-Sup1; *L* = 25) RNCs

The *E. coli* SecM stalling sequence in the ADR1a-SecM (*L* = 25) construct was modified by mutating five residues to obtain the Sup1 version of the *Mannheimia succiniciproducens* SecM AP (HPPIRGSP) (Yap and Bernstein, 2009), yielding ADR1a-SecM (*Ms*-Sup1; *L* = 25). The construct was subsequently cloned into the p7XNH vector. The final sequence used was MHHHHHHH HHHLEVLFQGPSYPYDVPDYAKPYPCGLCNRCFTRRDLLIRHAQKIHSGNSG SGVMSSFSTPVWISQHPPIRGSPA, including N-terminal His_{10}-CT-HA tags for purification.

In vitro translation in the presence of 50 μM $ZnCl_2$ was performed using 500 μl of the PURE system (NEB) following the manufacturer's instructions. RNCs were prepared as described in the Supplemental Information.

Cryo-EM Specimen Preparation, Data Collection, Processing, and Model Building

Carbon-coated holey grid preparation of ADR1a-SecM (*Ms*-Sup1; *L* = 25) RNCs was carried out as described previously (Bischoff et al., 2014). Cryo-EM data were collected on a Titan Krios TEM (FEI) operated at 300 keV and

equipped with a back-thinned Falcon II (FEI) direct electron detector, as described in the Supplemental Information.

All processing was performed using the SPIDER software package (Frank et al., 1996). The final data set contained 151,900 particles and was refined to a final average resolution of 4.8 Å according to the Fourier shell correlation criterion at a cutoff of 0.14.

For structural comparison and interpretation of the cryo-EM density obtained, we fitted the structure of the *E. coli* 70S ribosome (PDB: 3OFR) using UCSF Chimera (Pettersen et al., 2004). A poly-alanine model of the SecM-stalled nascent chain was built on the basis of the model of a TnaC stalled peptide (PDB: 4YU8) (Bischoff et al., 2014). The extra density at the end of the stalled SecM was compared with PDB-derived density maps of the ADR1a domain (PDB: 2ADR) at different resolutions and contour levels. Finally, the structure of the ADR1a domain was rigid-body-fitted according to the highest cross-correlation between the density model maps and the electron density.

ACCESSION NUMBERS

The electron microscopy map was deposited in the Electron Microscopy Data Bank under accession number EMD-3079.

SUPPLEMENTAL INFORMATION

Supplemental Information includes Supplemental Experimental Procedures and four figures and can be found with this article online at http://dx.doi.org/10.1016/j.celrep.2015.07.065.

AUTHOR CONTRIBUTIONS

G.v.H, R.B., E.P.O. and J.D.P. conceived the project. O.B.N., R.H., J.M., L.B., M.J. and A.M.-L. designed and performed the experiments. J.M., S.W., L.B., M.J., F.T., and E.P.O. performed the computational analyses. G.v.H wrote the manuscript with input from all other authors.

ACKNOWLEDGMENTS

This work was supported by grants from the Swedish Foundation for Strategic Research, the European Research Council (ERC-2008-AdG 232648), the Swedish Cancer Foundation, the Swedish Research Council, and the Knut and Alice Wallenberg Foundation to G.v.H; from the NIH (GM51266 and GM09968701) to J.D.P.; and by a grant from the Wenner-Gren Foundations to M.J. J.M. acknowledges an Early Postdoc Mobility Fellowship from the Swiss National Science Foundation and a Postdoc Fellowship granted by Novartis Foundation for Biomedical Research.

Received: June 17, 2015
Revised: July 17, 2015
Accepted: July 29, 2015
Published: August 27, 2015

REFERENCES

Bhushan, S., Gartmann, M., Halic, M., Armache, J.P., Jarasch, A., Mielke, T., Berninghausen, O., Wilson, D.N., and Beckmann, R. (2010). α-Helical nascent polypeptide chains visualized within distinct regions of the ribosomal exit tunnel. Nat. Struct. Mol. Biol. *17*, 313–317.

Bhushan, S., Hoffmann, T., Seidelt, B., Frauenfeld, J., Mielke, T., Berninghausen, O., Wilson, D.N., and Beckmann, R. (2011). SecM-stalled ribosomes adopt an altered geometry at the peptidyl transferase center. PLoS Biol. *9*, e1000581.

Bischoff, L., Berninghausen, O., and Beckmann, R. (2014). Molecular basis for the ribosome functioning as an L-tryptophan sensor. Cell Rep. *9*, 469–475.

Butkus, M.E., Prundeanu, L.B., and Oliver, D.B. (2003). Translocon "pulling" of nascent SecM controls the duration of its translational pause and secretion-responsive secA regulation. J. Bacteriol. *185*, 6719–6722.

Conti, B.J., Elferich, J., Yang, Z., Shinde, U., and Skach, W.R. (2014). Cotranslational folding inhibits translocation from within the ribosome-Sec61 translocon complex. Nat. Struct. Mol. Biol. *21*, 228–235.

Cymer, F., and von Heijne, G. (2013). Cotranslational folding of membrane proteins probed by arrest-peptide-mediated force measurements. Proc. Natl. Acad. Sci. U S A *110*, 14640–14645.

Cymer, F., Hedman, R., Ismail, N., and von Heijne, G. (2015). Exploration of the arrest peptide sequence space reveals arrest-enhanced variants. J. Biol. Chem. *290*, 10208–10215.

Frank, J., Radermacher, M., Penczek, P., Zhu, J., Li, Y., Ladjadj, M., and Leith, A. (1996). SPIDER and WEB: processing and visualization of images in 3D electron microscopy and related fields. J. Struct. Biol. *116*, 190–199.

Goldman, D.H., Kaiser, C.M., Milin, A., Righini, M., Tinoco, I., Jr., and Bustamante, C. (2015). Ribosome. Mechanical force releases nascent chain-mediated ribosome arrest in vitro and in vivo. Science *348*, 457–460.

Ismail, N., Hedman, R., Schiller, N., and von Heijne, G. (2012). A biphasic pulling force acts on transmembrane helices during translocon-mediated membrane integration. Nat. Struct. Mol. Biol. *19*, 1018–1022.

Ismail, N., Hedman, R., Lindén, M., and von Heijne, G. (2015). Charge-driven dynamics of nascent-chain movement through the SecYEG translocon. Nat. Struct. Mol. Biol. *22*, 145–149.

Johansson, M., Chen, J., Tsai, A., Kornberg, G., and Puglisi, J.D. (2014). Sequence-dependent elongation dynamics on macrolide-bound ribosomes. Cell Rep. *7*, 1534–1546.

Kaiser, C.M., Goldman, D.H., Chodera, J.D., Tinoco, I., Jr., and Bustamante, C. (2011). The ribosome modulates nascent protein folding. Science *334*, 1723–1727.

Kelkar, D.A., Khushoo, A., Yang, Z., and Skach, W.R. (2012). Kinetic analysis of ribosome-bound fluorescent proteins reveals an early, stable, cotranslational folding intermediate. J. Biol. Chem. *287*, 2568–2578.

Kolb, V.A., Makeyev, E.V., and Spirin, A.S. (2000). Co-translational folding of an eukaryotic multidomain protein in a prokaryotic translation system. J. Biol. Chem. *275*, 16597–16601.

Kosolapov, A., and Deutsch, C. (2009). Tertiary interactions within the ribosomal exit tunnel. Nat. Struct. Mol. Biol. *16*, 405–411.

Kowarik, M., Küng, S., Martoglio, B., and Helenius, A. (2002). Protein folding during cotranslational translocation in the endoplasmic reticulum. Mol. Cell *10*, 769–778.

Lin, K.F., Sun, C.S., Huang, Y.C., Chan, S.I., Koubek, J., Wu, T.H., and Huang, J.J. (2012). Cotranslational protein folding within the ribosome tunnel influences trigger-factor recruitment. Biophys. J. *102*, 2818–2827.

Marshall, R.A., Dorywalska, M., and Puglisi, J.D. (2008). Irreversible chemical steps control intersubunit dynamics during translation. Proc. Natl. Acad. Sci. U S A *105*, 15364–15369.

Mingarro, I., Nilsson, I., Whitley, P., and von Heijne, G. (2000). Different conformations of nascent polypeptides during translocation across the ER membrane. BMC Cell Biol. *1*, 3–10.

Nicola, A.V., Chen, W., and Helenius, A. (1999). Co-translational folding of an alphavirus capsid protein in the cytosol of living cells. Nat. Cell Biol. *1*, 341–345.

Nissley, D.A., and O'Brien, E.P. (2014). Timing is everything: unifying codon translation rates and nascent proteome behavior. J. Am. Chem. Soc. *136*, 17892–17898.

O'Brien, E.P., Hsu, S.T., Christodoulou, J., Vendruscolo, M., and Dobson, C.M. (2010). Transient tertiary structure formation within the ribosome exit port. J. Am. Chem. Soc. *132*, 16928–16937.

O'Brien, E.P., Christodoulou, J., Vendruscolo, M., and Dobson, C.M. (2011). New scenarios of protein folding can occur on the ribosome. J. Am. Chem. Soc. *133*, 513–526.

O'Brien, E.P., Christodoulou, J., Vendruscolo, M., and Dobson, C.M. (2012). Trigger factor slows co-translational folding through kinetic trapping while

sterically protecting the nascent chain from aberrant cytosolic interactions. J. Am. Chem. Soc. *134*, 10920–10932.

Párraga, G., Horvath, S.J., Eisen, A., Taylor, W.E., Hood, L., Young, E.T., and Klevit, R.E. (1988). Zinc-dependent structure of a single-finger domain of yeast ADR1. Science *241*, 1489–1492.

Pettersen, E.F., Goddard, T.D., Huang, C.C., Couch, G.S., Greenblatt, D.M., Meng, E.C., and Ferrin, T.E. (2004). UCSF Chimera—a visualization system for exploratory research and analysis. J. Comput. Chem. *25*, 1605–1612.

Rich, A.M., Bombarda, E., Schenk, A.D., Lee, P.E., Cox, E.H., Spuches, A.M., Hudson, L.D., Kieffer, B., and Wilcox, D.E. (2012). Thermodynamics of Zn^{2+} binding to Cys_2His_2 and $Cys_2HisCys$ zinc fingers and a Cys_4 transcription factor site. J. Am. Chem. Soc. *134*, 10405–10418.

Schwarz, D., Junge, F., Durst, F., Frölich, N., Schneider, B., Reckel, S., Sobhanifar, S., Dötsch, V., and Bernhard, F. (2007). Preparative scale expression of membrane proteins in Escherichia coli-based continuous exchange cell-free systems. Nat. Protoc. *2*, 2945–2957.

Shimizu, Y., Inoue, A., Tomari, Y., Suzuki, T., Yokogawa, T., Nishikawa, K., and Ueda, T. (2001). Cell-free translation reconstituted with purified components. Nat. Biotechnol. *19*, 751–755.

Shimizu, Y., Kanamori, T., and Ueda, T. (2005). Protein synthesis by pure translation systems. Methods *36*, 299–304.

Sohmen, D., Chiba, S., Shimokawa-Chiba, N., Innis, C.A., Berninghausen, O., Beckmann, R., Ito, K., and Wilson, D.N. (2015). Structure of the *Bacillus subtilis* 70S ribosome reveals the basis for species-specific stalling. Nat. Commun. *6*, 6941.

Sugita, Y., and Okamoto, Y. (1999). Replica-exchange molecular dynamics method for protein folding. Chem. Phys. Lett. *314*, 141–151.

Tsai, A., Kornberg, G., Johansson, M., Chen, J., and Puglisi, J.D. (2014). The dynamics of SecM-induced translational stalling. Cell Rep. *7*, 1521–1533.

Tu, L., Khanna, P., and Deutsch, C. (2014). Transmembrane segments form tertiary hairpins in the folding vestibule of the ribosome. J. Mol. Biol. *426*, 185–198.

Voss, N.R., Gerstein, M., Steitz, T.A., and Moore, P.B. (2006). The geometry of the ribosomal polypeptide exit tunnel. J. Mol. Biol. *360*, 893–906.

Waudby, C.A., Launay, H., Cabrita, L.D., and Christodoulou, J. (2013). Protein folding on the ribosome studied using NMR spectroscopy. Prog. Nucl. Magn. Reson. Spectrosc. *74*, 57–75.

Welte, T., Kudva, R., Kuhn, P., Sturm, L., Braig, D., Müller, M., Warscheid, B., Drepper, F., and Koch, H.G. (2012). Promiscuous targeting of polytopic membrane proteins to SecYEG or YidC by the Escherichia coli signal recognition particle. Mol. Biol. Cell *23*, 464–479.

Wheelan, S.J., Marchler-Bauer, A., and Bryant, S.H. (2000). Domain size distributions can predict domain boundaries. Bioinformatics *16*, 613–618.

Yap, M.N., and Bernstein, H.D. (2009). The plasticity of a translation arrest motif yields insights into nascent polypeptide recognition inside the ribosome tunnel. Mol. Cell *34*, 201–211.

Zhou, J., Lancaster, L., Trakhanov, S., and Noller, H.F. (2012). Crystal structure of release factor RF3 trapped in the GTP state on a rotated conformation of the ribosome. RNA *18*, 230–240.

Cell Reports

Supplemental Information

Cotranslational Protein Folding inside the Ribosome Exit Tunnel

Ola B. Nilsson, Rickard Hedman, Jacopo Marino, Stephan Wickles, Lukas Bischoff, Magnus Johansson, Annika Müller-Lucks, Fabio Trovato, Joseph D. Puglisi, Edward P. O'Brien, Roland Beckmann, and Gunnar von Heijne

a

MANRSFIYEPFQIPSGSMMPTLNSTDFILVEKFAYGIKDPIYQKTLIETGHPKRGDIVVFKYPEDPKLDYIKRAVGLPGDKVTY
DPVSKELTIQPGCSSGQACENALPVTYSNVEPSDFVQTFSRRNGGEATSGFFEVPKQETKENGIRLSETSGSGSKPYPCGLCNR
CFTRRDLLIRHAQKIHSGNSGSGVPGQQNATWIVPPGQYFMMGDWMSSFSTPVWISQAQGIRAGPGSSDKQEGEWPTGLRLSRI
GGIH

b

Ribosome (~30 aa)

Sequence	*L*
GSGSKPYPCGLCNRCFTRRDLLIRHAQKIHSGNFSTPVWISQAQGIRAGP	17
GSGSKPYPCGLCNRCFTRRDLLIRHAQKIHSGNMSSFSTPVWISQAQGIRAGP	20
GSGSKPYPCGLCNRCFTRRDLLIRHAQKIHSGNSGMSSFSTPVWISQAQGIRAGP	22
GSGSKPYPCGLCNRCFTRRDLLIRHAQKIHSGNSGSMSSFSTPVWISQAQGIRAGP	23
GSGSKPYPCGLCNRCFTRRDLLIRHAQKIHSGNSGSGMSSFSTPVWISQAQGIRAGP	24
GSGSKPYPCGLCNRCFTRRDLLIRHAQKIHSGNSGSGVMSSFSTPVWISQAQGIRAGP	25
GSGSKPYPCGLCNRCFTRRDLLIRHAQKIHSGNSGSGVPMSSFSTPVWISQAQGIRAGP	26
GSGSKPYPCGLCNRCFTRRDLLIRHAQKIHSGNSGSGVPGMSSFSTPVWISQAQGIRAGP	27
GSGSKPYPCGLCNRCFTRRDLLIRHAQKIHSGNSGSGVPGWMSSFSTPVWISQAQGIRAGP	28
GSGSKPYPCGLCNRCFTRRDLLIRHAQKIHSGNSGSGVPGQWMSSFSTPVWISQAQGIRAGP	29
GSGSKPYPCGLCNRCFTRRDLLIRHAQKIHSGNSGSGVPGQQWMSSFSTPVWISQAQGIRAGP	30
GSGSKPYPCGLCNRCFTRRDLLIRHAQKIHSGNSGSGVPGQQNWMSSFSTPVWISQAQGIRAGP	31
GSGSKPYPCGLCNRCFTRRDLLIRHAQKIHSGNSGSGVPGQQNAWMSSFSTPVWISQAQGIRAGP	32
GSGSKPYPCGLCNRCFTRRDLLIRHAQKIHSGNSGSGVPGQQNATWMSSFSTPVWISQAQGIRAGP	33
GSGSKPYPCGLCNRCFTRRDLLIRHAQKIHSGNSGSGVPGQQNATWAIWMSSFSTPVWISQAQGIRAGP	36
GSGSKPYPCGLCNRCFTRRDLLIRHAQKIHSGNSGSGVPGQQNATWIVPWMSSFSTPVWISQAQGIRAGP	37
GSGSKPYPCGLCNRCFTRRDLLIRHAQKIHSGNSGSGVPGQQNATWIVPPGWMSSFSTPVWISQAQGIRAGP	39
GSGSKPYPCGLCNRCFTRRDLLIRHAQKIHSGNSGSGVPGQQNATWIVPPGQYWMSSFSTPVWISQAQGIRAGP	41
GSGSKPYPCGLCNRCFTRRDLLIRHAQKIHSGNSGSGVPGQQNATWIVPPGQYFMWMSSFSTPVWISQAQGIRAGP	43
GSGSKPYPCGLCNRCFTRRDLLIRHAQKIHSGNSGSGVPGQQNATWIVPPGQYFMMGDWMSSFSTPVWISQAQGIRAGP	46

Figure S1, related to Fig. 1. (a) Seqeunce of the full ADR1a-SecM(*L*=46) construct. ADR1a is in red, the SecM AP in green, residues from the periplasmic domain of *E. coli* LepB in black, and linker residues generated during construction in grey. (b) Sequences of the ADR1a-AP part of all constructs analyzed in Fig. 1d. Zn^{2+}-binding residues are highligthed in blue.

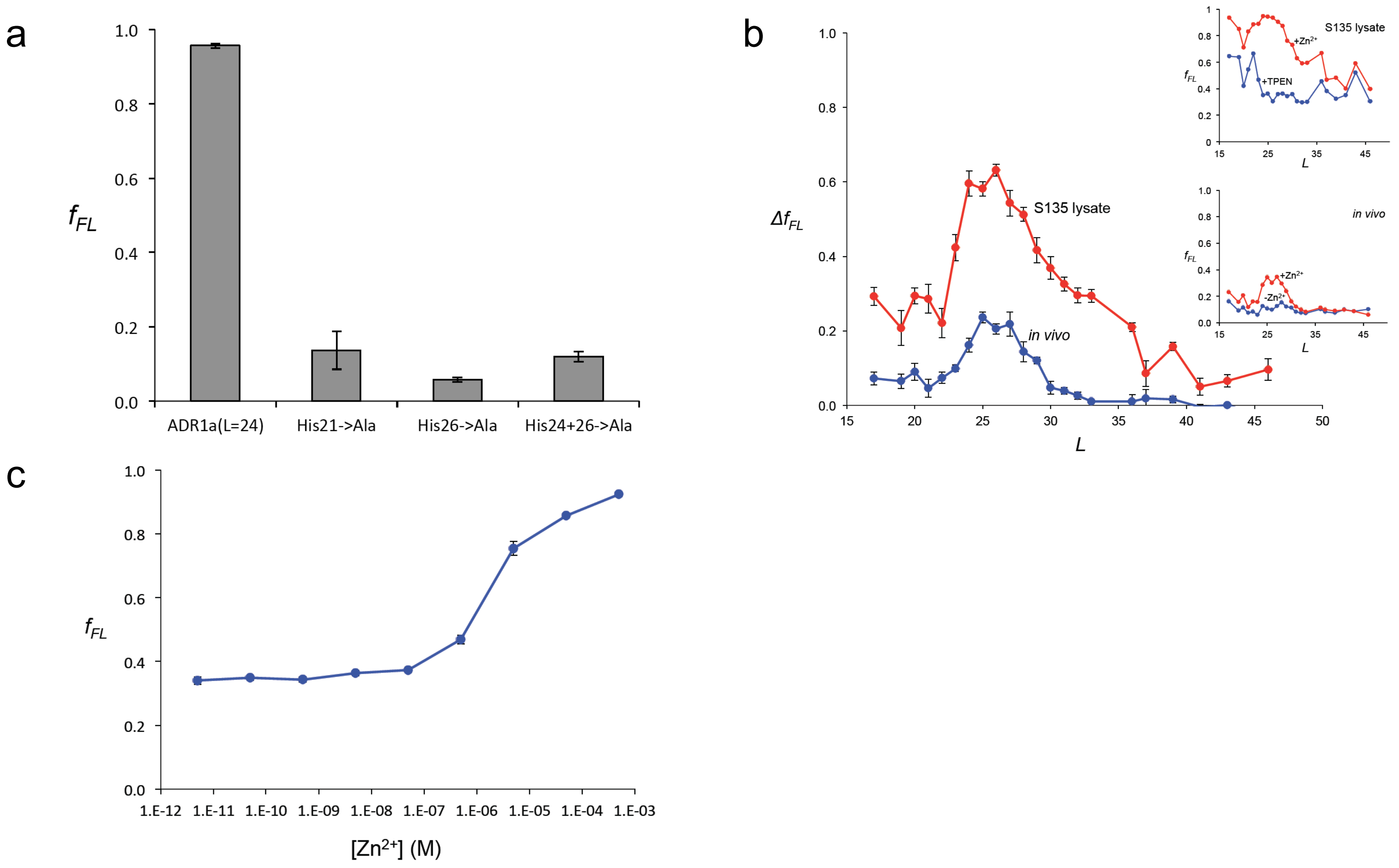

Figure S2, related to Fig.1. (a) Mutation of the two Zn^{2+}-binding His residues in the ADR1a-SecM(*L*=24) construct to Ala reduces f_{FL} to background values. Standard errors (s.e.m.) are indicated. (b) Zn^{2+}-induced folding of ADR1a in an *E.coli* S135 lysate (red) and *in vivo* (blue). The difference in f_{FL} between reactions run in the presence (500 µM) and absence of Zn^{2+} is shown. Standard errors (s.e.m.) are indicated. Insets show the individual f_{FL} profiles $\pm Zn^{2+}$ for the S135 lysate (top) and *in vivo* (bottom). (c) Zn^{2+} titration of folding of ADR1a(*L*=26) translated in *E. coli* S135 lysate. Standard errors (s.e.m.) are indicated.

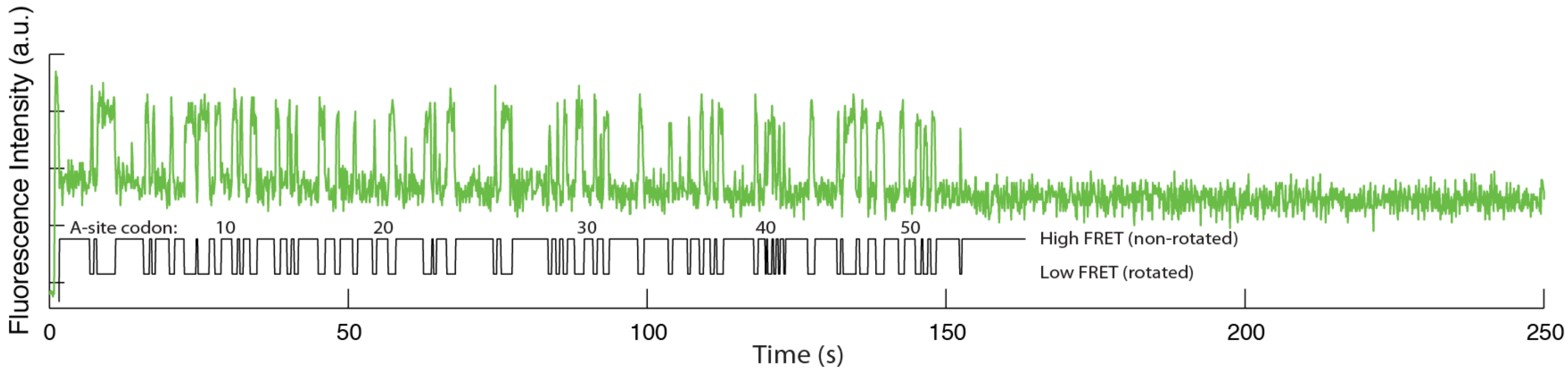

Figure S3, related to Fig.2. Time-trace showing one ribosome translating the ADR1a-SecM ORF in the absence of Zn^{2+}. The ribosome stalls at codon 54.

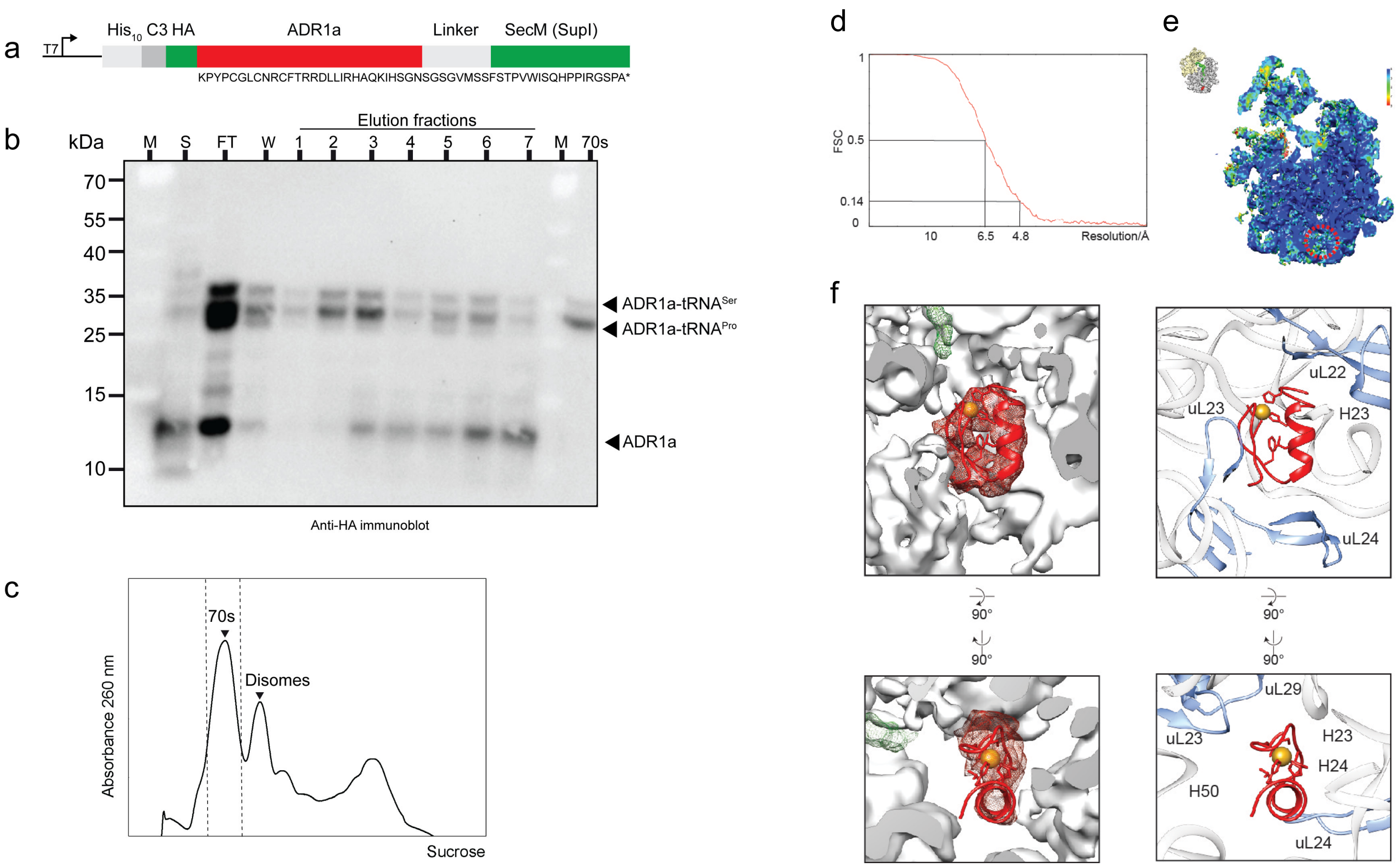

Figure S4, related to Fig.4. Purification of the ADR1a-SecM(*Ms*-Sup1; *L*=25) ribosome-nascent chain complex, and resolution of ADR1-RNCs and local environment of the ADR1 domain in the ribsomal tunnel. (a) Construct for programming and affinity purification of ADR1a-SecM(*Ms*-Sup1; *L*=25) RNCs for cryo-EM analysis. (b) Analysis of RNC purification by Western-blot using anti-HA antibody and 12% Nu-Page gel separation of the supernatant fraction after ultracentrifugation (S), the flow through fraction after Ni-NTA column (FT), the wash fraction (W), and elution fractions. Bands corresponding to Ser- and Pro-peptidyl-tRNA-ADR1a complexes and the free peptide corresponding to ADR1a are indicated. (c) UV profile after sucrose density gradient centrifugation of affinity purified RNCs. Dashed lines indicate the fraction collected for by cryo-EM analysis. (d) FSC of the RNC-ADR1a-SecM(*Ms*-Sup1;*L*=25) reconstruction indicating an average resolution of 4.8 Å. (e) Local resolution (Kucukelbir et al., 2014) shown on a cut density, revealing resolution for the ADR1a domain similar to the ribosome. (f) Close-up of the tunnel with the structure of the ADR1a domain fitted as a rigid body depicted in red. The identities of nearby ribosomal proteins and rRNA are indicated.

SUPPLEMENTAL EXPERIMENTAL PROCEDURES

Enzymes and Chemicals

Unless otherwise stated, all enzymes were obtained from Thermo Scientific (Waltham, MA, USA) and New England Biolabs (Ipswich, MA, USA). Oligonucleotides were from Eurofins MWG Operon (Ebersberg, Germany). PUREfrex™ cell-free translation system was purchased from BioNordika (Stockholm, Sweden) and DNA/RNA purification kits were obtained from Qiagen (Hilden, Germany). [^{35}S]-methionine was from PerkinElmer (Waltham, MA, USA). All other reagents were from Sigma-Aldrich (St. Louis, MO, USA).

DNA Manipulations

All ADR1a constructs were generated from the previously described pING1 plasmid carrying a truncated *lepB* gene containing a [6L,13A] H segment insert and the *Escherichia coli* SecM arrest peptide, FSTPVWISQAQGIRAGP, under the control of an arabinose-inducible promoter (Ismail et al., 2012). A soluble, non-membrane targeted LepB derivative was generated by a deletion of codon 4-77 using PCR, corresponding to the removal of transmembrane segment 1 and 2. The resulting plasmid was digested with *SpeI* and *KpnI* to release the [6L,13A] segment, and oligonucleotides corresponding to a GSGS-flanked ADR1a domain (GSGS-KPYPCGLCNRCFTRRDLLIRHAQKIHSGN-SGSG) were ligated in its place. Shorter linker lengths, *L*, between ADR1a and the arrest peptide were generated by shortening the linker from its N-terminal end by PCR as previously described (Ismail et al., 2012). Site-directed mutagenesis was performed to generate constructs with the non-functional FSTPVWISQAQGIRAG<u>A</u> arrest peptide and constructs with ADR1a domains where either one or both of the underlined, Zn^{2+}-binding His residues in the

sequence KPYPCGLCNRCFTRRDLLIRHAQKIHSGN were changed to Ala. For RNA transcription using the T7 promoter, all constructs were subcloned into pET19b (Novagen, Madison, WI, USA) using *NcoI* and *BamHI*.

***In Vitro* Transcription and Translation for Measurements of f_{FL}.**

In vitro transcription was performed with T7 RNA polymerase according to the manufacturer's protocol (Promega) using PCR products as templates for the generation of truncated nascent chains. RNA obtained was purified using RNeasy Mini Kit (Qiagen). Translation was performed in the commercially available PUREfrex™ system (Shimizu et al., 2005) and in a S135 *E. coli* extract. The S135 cell extract was prepared as previously described (Schwarz et al., 2007). To obtain an essentially Zn^{2+}-free S135 cell extract for the Zn^{2+}-titration assay (Fig. S2c), we added three additional dialysis steps to the normal protocol, where 50 µM of the Zn^{2+} chelator N,N,N,N-Tetrakis(2-pyridylmethyl)ethylenediamine (TPEN) was added to the normal dialysis buffer. *In vitro* translation in the PURE system was performed according to the manufacturer's protocol. *In vitro* translation in the S135 cell extracts was performed essentially as described (Welte et al., 2012). Translation was carried out in a buffer containing 40 mM Tris pH 7.5, 140 mM KOAc, 10 mM Mg^{2+} and 0.8 mM Spermidine. The translation reactions were supplemented with either 50 µM (PURE) or 500 µM (S135 extract) zinc acetate, or 50 µM TPEN. Synthesis of [^{35}S]-Met labeled polypeptides in both translation systems was performed at 37 °C with shaking at 500 rpm for 15 minutes. The reaction was stopped by the addition of equal volume of 10% ice-cold TCA and the samples were incubated on ice for 30 min and spun for 5 min at 20,800 g at 4°C. Pellets were dissolved in sample buffer and treated with RNase A (400 µg ml^{-1}) for 15 min at 37 °C before the samples were resolved by SDS-PAGE. Gels were visualized using a Fuji FLA-3000 phosphoimager and the

ImageGauge V4.23 software. Quantification of protein bands was performed using the QtiPlot 0.9.7.10 software. f_{FL} values were calculated as $f_{FL} = I_{FL}/(I_{FL}+I_A)$, where I_{FL} is the intensity of the band corresponding to the full-length protein and I_A is the intensity of the band corresponding to the arrested form of the protein, *c.f.*, Fig. 1c. Experiments were repeated three times, and standard errors (s.e.m.) were calculated.

***In Vivo* Pulse-Labeling Analysis**

E. coli MC1061 cells bearing the respective plasmids were grown overnight at 37°C in M9 minimal media supplemented with 19 natural amino acids (1 μg ml^{-1}; no Met), 100 μg ml^{-1} thiamine, 0.1 mM $CaCl_2$, 2 mM $MgSO_4$, 0.4% (w/v) fructose, and 100 μg ml^{-1} ampicillin. Cultures were back-diluted to OD_{600} = 0.1, grown to OD_{600} = 0.35 and split into two subcultures. Expression of the ADR1a-SecM constructs was induced with 0.2% (w/v) arabinose for 5 min. $ZnCl_2$ was added to one of the two subcultures to a final concentration of 0.5 mM at the point of induction using a mixture of arabinose and $ZnCl_2$. Cells were then pulsed-labeled with [^{35}S]-Met for 2 min at 37°C before being added to an equal volume of 20% ice-cold trichloroacetic acid (TCA). Samples were incubated on ice for 30 min and spun for 5 min at 20,800 g at 4°C. Pellets were washed with cold acetone, spun again for 5 min at 4°C, and subsequently solubilized in Tris-SDS solution (10 mM Tris-Cl, pH 7.5, 2% SDS) at 95°C for 10 min. Samples were spun for 5 min at room temperature and the lysate was used for immunoprecipitation using LepB antisera. The samples were resolved by SDS-PAGE and quantitated as described above. Experiments were repeated three times using independent culture incubations, and standard errors (s.e.m.) were calculated.

Single-Ribosome Inter-Subunit FRET Experiments.

fMet-tRNA^{fMet} bound 30S pre-initiated complexes (PICs), Cy3B labeled on the 16S rRNA (Marshall et al., 2008), were formed on the ADR1a-SecM(*L*=24; Δ1-158)

mRNA constructs and immobilized to the surface of pre-treated zero-mode waveguide (ZMW) chips (SMRT Cell, *Pacific Biosciences*) through hybridization of the mRNAs to biotinylated splint DNA oligos (Tsai et al., 2014). Elongation mixtures, containing 200 nM fluorescence-quencher-labeled (BHQ-2) 50S ribosomal subunits, 240 nM EF-G, and 3 μM total aa-tRNA·EF-Tu·GTP ternary complex (TC), were delivered to the ZMW chips in a modified PacBio RS sequencer where all individual ZMWs are illuminated with 532 nm laser and fluorescence data is acquired over time (Tsai et al., 2014). Preparation of native or fluorophore-labeled biomolecules was performed as described in (Johansson et al., 2014) and references therein. The elongation reactions were carried out in a Tris-based polymix buffer at 20°C in the presence of 1 μM IF2, 4 mM GTP, 2 mM Trolox and a PCA/PCD oxygen-scavenging system. Fluorescence data was collected at 10 Hz for 10 min, and filtered and analyzed using MATLAB (MathWorks) scripts as has been described previously (Tsai et al., 2014). ZMW chips were loaded stochastically at 30 % occupancy. The 30 % ZMWs with lowest signal were used to calculate background and σ. The wells with signal greater than n*σ above background, that lasted longer than 10 s, were selected for n = N[1 10]. The minimal of discrete differential of the resulting function (number of picked wells from n) was used to identify the best n value for molecule identification. The picked traces were manually curated for fluorescence intensity, fluorescence lifetime, and the fluorescence intensity change when the laser is turned on, to assure single occupancy of ribosomal subunits in the wells. Intersubunit FRET states were assigned based on a hidden Markov model approach (McKinney et al., 2006) with manual correction, from productive traces showing stable [Cy3B]30S immobilization, joining of 50S (yielding high-FRET), and at least two high-low-high-FRET cycles signaling intersubunit rotation (Tsai et al.,

2014). Average state lifetimes were calculated by fitting the individual lifetimes to a single-exponential distribution using maximum-likelihood parameter estimation. Only lifetimes from productive states were included (i.e., low-FRET states that was followed by a high-FRET state and vice versa) to eliminate artifacts from photophysical effects. The density of elongating ribosomes, decreasing with codon number due to both photobleaching and erroneous translation termination, was calculated and summarized from assigned FRET states in n individual traces.

Molecular Dynamics Simulations

The cotranslational folding curve of the ribosome-ADR1a nascent chain complex was calculated on an arrested ribosome using the coarse-grained model of O'Brien and co-workers (O'Brien et al., 2011, 2012) in which amino-acids are represented as one interaction site, purine containing nucleotides as three interaction sites and pyrimidine containing nucleotides as four interaction sites. In this model electrostatic interactions are treated using Debye-Huckel theory, with a 10 Å Debye screening length. We utilized the force-field and Langevin Dynamics protocol published previously (O'Brien et al., 2011, 2012). Briefly, a structure-based force-field (Ueda et al., 1978; Onuchic and Wolynes, 2004) was used for the ADR1a zinc finger domain (PDB ID: 2ADR) and a transferable force field (O'Brien et al., 2011) was used for the unstructured linkers. In the simulations, the 50S subunit of the *E. coli* ribosome (PDB ID: 3UOS) and the nascent chain are explicitly represented. Zinc ions were not represented in the simulation, instead their effect on protein stability was implicitly accounted for by linearly scaling the Lennard-Jones well-depth of residue pairs that are in contact in the native state (O'Brien et al., 2011, 2012) such that the stability of the folded zinc-finger in isolation was equal to -2.0 kcal/mol at 310 K. ADR1a was then covalently attached to unstructured linkers having the same sequences as used in

the experiments (see Fig. S1). Linker lengths of 17 to 46 residues were simulated. At each linker length, replica-exchange simulations (Sugita and Okamoto, 1999) were run with 8 temperature windows ranging between 290 and 370 K. A simulation structure of the zinc finger domain was classified as folded if its root-mean-squared deviation (RMSD) from the ADR1a NMR structure was < 3.5 Å, and classified as unfolded if the RMSD > 5.5 Å. The WHAM equations (Kumar et al., 1992) were then utilized to calculate the probability of the domain being folded as a function of linker length at 310 K.

Cloning and Purification of ADR1a-SecM(*Ms*-Sup1, *L*=25) Ribosome-Nascent Chain Complexes

The ADR1a-SecM(*L*=25) construct, which is at the peak of the force profile in Fig. 1d, was chosen for large-scale preparation and cryo-EM analysis. The *E. coli* SecM stalling sequence was modified by mutating 5 residues to obtain the Sup1 version of the *M. succiniproducens* SecM AP (HPPIRGSP) (Yap and Bernstein, 2009). The resulting sequence was overlapped by PCR to the DNA fragment encoding the last 29 amino acids of the yeast ADR1a protein, yielding ADR1a-SecM(*Ms*-Sup1; *L*=25), Fig. S4a. Primers containing 5′ SapI sites were used to PCR-amplify ADR1a-SecM(*Ms*-Sup1; *L*=25), which was subsequently cloned into a p7XNH vector by using the FX cloning method (Geertsma, 2014). 5 ml of the FX cloning reaction were used to transform chemically competent *E. coli* MC1061 cells. Single colonies were further used to prepare plasmids that were sequence verified and used for *in vitro* translation experiments. The final sequence used was MHHHHHHHHHHLEVLFQGPSYPYDVPDYAKPYPCGLCNR CFTRRDLLIRHAQKIHSGNSGSGVMSSFSTPVWISQHPPIRGSPA, including N-terminal His_{10}-CT-HA tags for purification.

In vitro translation was performed by using the PURE System (NEB) following the manufacturer's instructions. A 500 µl PURE System reaction was incubated for 90 minutes at 37°C, in presence of 50 µM $ZnCl_2$. Ribosomes were then pelleted trough a sucrose-cushion (50 mM Hepes pH 7.2, 250 mM KOAc, 25 mM $Mg[OAc]_2$, 75 mM sucrose) for 1 hour at 100.000 x g at 4 °C. The ribosomal pellet was resuspended in equilibration buffer (50 mM Hepes pH 7.4, 500 mM KOAc, 25 mM $Mg[OAc]_2$, 0.03% DDM, 50 µM $ZnCl_2$, 125 mM sucrose) and incubated with 400 ml Ni-NTA resin (Protino, Macheray-Nagel, Germany) for 1 hour at 4°C. The resin was subsequently washed with ten column volumes (CV) of 50mM Hepes pH 7.4, 500 mM KOAc, 25 mM $Mg[OAc]_2$, 0.03% DDM, 50 µM $ZnCl_2$, 125 mM sucrose, 20 mM imidazole. Ribosome nascent-chain complexes (RNCs) were eluted in 0.5 CV fractions with 50 mM Hepes pH 7.2 , 500 mM KOAc, 25 mM $Mg[OAc]_2$, 0.03% DDM, 50 µM $ZnCl_2$, 125 mM sucrose, 300 mM imidazole. The elution fractions were subsequently loaded onto a 10-40% density sucrose gradient and centrifuged at 45.000 rpm in a SW40 rotor (Beckman Coulter) for three hours. The 70s peak was collected and RNCs were concentrated by ultracentrifugation (100.000xg for 1 hour). The ribosome pellet was finally resuspended in cryo-grid buffer (20 mM Hepes pH 7.2 , 50 mM KOAc, 5 mM $Mg[OAc]_2$, 0.03% DDM, 50 µM $ZnCl_2$, 125 mM sucrose) to a final $O.D._{260} = 6$, aliquoted in small volumes, and finally stored at -80°C until needed for the preparation of cryo-EM grids. Correct size of the RNC-complexes were verified by 12% Nu-Page (Life Technologies Inc.), and gels were submitted to semidry electroblotting and immunodetection with a primary antibody raised against a HA-tag (Roche). Chemiluminescence detection was done by using the SuperSignal West Dura Extended Duration Substrate (Thermo Scientific) and the Fujifilm LAS-3000 imaging system.

Cryo-EM Specimen Preparation, Data Collection, Processing, and Model Building

Carbon-coated holey grid preparation of ADR1a-SecM(*Ms*-Sup1; *L*=25) RNCs was carried out as described previously (Bischoff et al., 2014). Cryo-EM data was collected on a Titan Krios TEM (FEI, USA) operated at 300 keV and equipped with a back-thinned Falcon II (FEI, USA) direct electron detector. The camera was calibrated for a nominal magnification of 75,000x, resulting in a pixel size of 1.37 Å at the specimen. Seven blocks of frames s^{-1} were recorded in automatic mode with a dose of 5 $e^{-}/Å^{2}$ per block at defocus values between -1 and -3.2 mm. Frames were aligned using the software developed by the Yifang Cheng lab at UCSF (Li et al., 2013).

Micrographs showing drift or contamination were manually discarded from the dataset. All processing was performed using the SPIDER software package (Frank et al., 1996). The initial dataset of 496,340 particles was first cleaned from non-ribosomal particles (306,243 ribosomal particles left) and subsequently sorted for the presence of A, P and E site tRNAs. The dataset that contained strong density for tRNA in the P-site, was further refined by applying a cross correlation cut-off. The final dataset contained 151,900 particles and was refined to a final average resolution of 4.8 Å according to the FSC criterion at cut-off at 0.14. Potential over-fitting was excluded by truncating high frequencies (low-pass filter at 8 Å) during the whole refinement process (Scheres and Chen, 2012).

For structural comparison and interpretation of the cryo-EM density obtained, we fitted the structure of the *E. coli* 70S ribosome (PDB ID: 3OFR), using UCSF Chimera (Pettersen et al., 2004). A poly-alanine model of the SecM-stalled nascent chain was built based on the model of a TnaC stalled peptide (PDB ID: 4YU8)

(Bischoff et al., 2014). The extra density at the end of the stalled SecM was extracted and compared with PDB-derived density maps of the ADR1a domain (PDB ID: 2ADR) at different resolutions and contour levels. Finally, the structure of the ADR1a domain was rigid-body fitted according to the highest cross-correlation between the density model maps and the electron density.

SUPPLEMENTAL REFERENCES

Bischoff, L., Berninghausen, O., and Beckmann, R. (2014). Molecular basis for the ribosome functioning as an L-tryptophan sensor. Cell reports *9*, 469-475.

Frank, J., Radermacher, M., Penczek, P., Zhu, J., Li, Y., Ladjadj, M., and Leith, A. (1996). SPIDER and WEB: processing and visualization of images in 3D electron microscopy and related fields. Journal of structural biology *116*, 190-199.

Geertsma, E.R. (2014). FX cloning: a simple and robust high-throughput cloning method for protein expression. Methods Mol Biol *1116*, 153-164.

Ismail, N., Hedman, R., Schiller, N., and von Heijne, G. (2012). A biphasic pulling force acts on transmembrane helices during translocon-mediated membrane integration. Nature Struct Molec Biol *19*, 1018-1022.

Johansson, M., Chen, J., Tsai, A., Kornberg, G., and Puglisi, J.D. (2014). Sequence-dependent elongation dynamics on macrolide-bound ribosomes. Cell reports *7*, 1534-1546.

Kumar, S., Rosenberg, J.M., Bouzida, D., Swendsen, R.H., and Kollman, P.A. (1992). THE weighted histogram analysis method for free-energy calculations on biomolecules. I. The method. J Comput Chem *13*, 1011-1021.

Li, X., Mooney, P., Zheng, S., Booth, C.R., Braunfeld, M.B., Gubbens, S., Agard, D.A., and Cheng, Y. (2013). Electron counting and beam-induced motion

correction enable near-atomic-resolution single-particle cryo-EM. Nat Methods *10*, 584-590.

Marshall, R.A., Dorywalska, M., and Puglisi, J.D. (2008). Irreversible chemical steps control intersubunit dynamics during translation. Proc Natl Acad Sci U S A *105*, 15364-15369.

McKinney, S.A., Joo, C., and Ha, T. (2006). Analysis of single-molecule FRET trajectories using hidden Markov modeling. Biophys J *91*, 1941-1951.

O'Brien, E.P., Christodoulou, J., Vendruscolo, M., and Dobson, C.M. (2011). New scenarios of protein folding can occur on the ribosome. J Am Chem Soc *133*, 513-526.

O'Brien, E.P., Christodoulou, J., Vendruscolo, M., and Dobson, C.M. (2012). Trigger factor slows co-translational folding through kinetic trapping while sterically protecting the nascent chain from aberrant cytosolic interactions. J Am Chem Soc *134*, 10920-10932.

Onuchic, J.N., and Wolynes, P.G. (2004). Theory of protein folding. Curr Opin Struct Biol *14*, 70-75.

Pettersen, E.F., Goddard, T.D., Huang, C.C., Couch, G.S., Greenblatt, D.M., Meng, E.C., and Ferrin, T.E. (2004). UCSF Chimera--a visualization system for exploratory research and analysis. Journal of computational chemistry *25*, 1605-1612.

Scheres, S.H., and Chen, S. (2012). Prevention of overfitting in cryo-EM structure determination. Nat Methods *9*, 853-854.

Schwarz, D., Junge, F., Durst, F., Frölich, N., Schneider, B., Reckel, S., Sobhanifar, S., Dötsch, V., and Bernhard, F. (2007). Preparative scale expression of

membrane proteins in Escherichia coli-based continuous exchange cell-free systems. Nat Protoc *2*, 2945-2957.

Shimizu, Y., Kanamori, T., and Ueda, T. (2005). Protein synthesis by pure translation systems. Methods *36*, 299-304.

Sugita, Y., and Okamoto, Y. (1999). Replica-exchange molecular dynamics method for protein folding. Chem Phys Lett *314*, 141-151.

Tsai, A., Kornberg, G., Johansson, M., Chen, J., and Puglisi, J.D. (2014). The dynamics of SecM-induced translational stalling. Cell reports *7*, 1521-1533.

Ueda, Y., Taketomi, H., and Gō, N. (1978). Studies on protein folding, unfolding, and fluctuations by computer simulation. II. A. Three-dimensional lattice model of lysozyme. Biopolymers *17*, 1531–1548.

Welte, T., Kudva, R., Kuhn, P., Sturm, L., Braig, D., Muller, M., Warscheid, B., Drepper, F., and Koch, H.G. (2012). Promiscuous targeting of polytopic membrane proteins to SecYEG or YidC by the Escherichia coli signal recognition particle. Mol Biol Cell *23*, 464-479.

Yap, M.N., and Bernstein, H.D. (2009). The plasticity of a translation arrest motif yields insights into nascent polypeptide recognition inside the ribosome tunnel. Mol Cell *34*, 201-211.
